# Metagenomic profiles of free-living archaea, bacteria and small eukaryotes in coastal areas of Sichang island, Thailand

**DOI:** 10.1186/1471-2164-13-S7-S29

**Published:** 2012-12-07

**Authors:** Naraporn Somboonna, Anunchai Assawamakin, Alisa Wilantho, Sithichoke Tangphatsornruang, Sissades Tongsima

**Affiliations:** 1 Department of Microbiology, Faculty of Science, Chulalongkorn University, Bangkok 10330, Thailand; 2 Genome Institute, National Center for Genetic Engineering and Biotechnology, Klong 1, Klong Luang, Pathum thani 12120, Thailand

## Abstract

**Background:**

Tha Wang and Tham Phang coasts, though situated at similar oceanographic positions on Sichang island, Chonburi province, Thailand, are different in bay geography and amount of municipal disturbances. These affect the marine ecosystems. The study used metagenomics combined with 16S and 18S rDNA pyrosequencing to identify types and distributions of archaea, bacteria, fungi and small eukaryotes of sizes ranges 0.45 and ~30 μm.

**Results:**

Following the open bay geography and minimal municipal sewages, Tham Phang coast showed the cleaner water properties, described by color, salinity, pH, conductivity and percent dissolved oxygen. The 16S and 18S rDNA metagenomic profiles for Tha Wang and Tham Phang coasts revealed many differences, highlighting by low Lennon and Yue & Clayton theta similarity indices (66.03-73.03% for 16S rDNA profiles, 2.85-25.38% for 18S rDNA profiles). For 16S rDNA, the percent compositions of species belonging to Proteobacteria, Bacteroidetes, Cyanobacteria, Firmicutes, Verrucomicrobia, Gammatimonadetes, Tenericutes, Acidobacteria, Spirochaetes, Chlamydiae, Euryarchaeota, Nitrospirae, Planctomycetes, Thermotogae and Aquificae were higher or distinctly present in Tha Wang. In Tham Phang, except Actinobacteria, the fewer number of prokaryotic species existed. For 18S rDNA, fungi represented 74.745% of the species in Tha Wang, whereas only 6.728% in Tham Phang. Basidiomycota (71.157%) and Ascomycota (3.060%) were the major phyla in Tha Wang. Indeed, Tha Wang-to-Tham Phang percent composition ratios for fungi Basidiomycota and Chytridiomycota were 1264.701 and 25.422, respectively. In Tham Phang, Brachiopoda (lamp shells) and Mollusca (snails) accounted for 80.380% of the 18S rDNA species detected, and their proportions were approximately tenfold greater than those in Tha Wang. Overall, coastal Tham Phang comprised abundant animal species.

**Conclusions:**

Tha Wang contained numerous archaea, bacteria and fungi, many of which could synthesize useful biotechnology gas and enzymes that could also function in high-saline and high-temperature conditions. Tham Phang contained less abundant archaea, bacteria and fungi, and the majority of the extracted metagenomes belonged to animal kingdom. Many microorganisms in Tham Phang were essential for nutrient-recycling and pharmaceuticals, for instances, *Streptomyces, Pennicilium *and *Saccharomyces*. Together, the study provided metagenomic profiles of free-living prokaryotes and eukaryotes in coastal areas of Sichang island.

## Background

Thailand situates around an equator, between 23.5 degree north and 23.5 degree south, causing the climate to be hot and rainy, which enhances the biodiversity of microorganisms. In addition to factors by sunlight, wind and tidal ranges, coastal niche represents areas where human disturbances are most situated, and is where land and sea meet with high influences by the bay characteristics. All these factors could affect types and distribution patterns of aquatic microorganisms and organisms [1-4]. Indeed, previous studies reported different proportions of organisms between Tha Wang and Tham Phang coasts of Sichang island, Thailand, and suggested the differences involved their differences in coastal quality (S. Piyatiratitivorakul and S. Rungsupa, personal communications). Nevertheless, no culture-independent study for inclusive databases on free-living microorganisms had been conducted in Tha Wang and Tham Phang coasts of Sichang.

**Figure 1:**
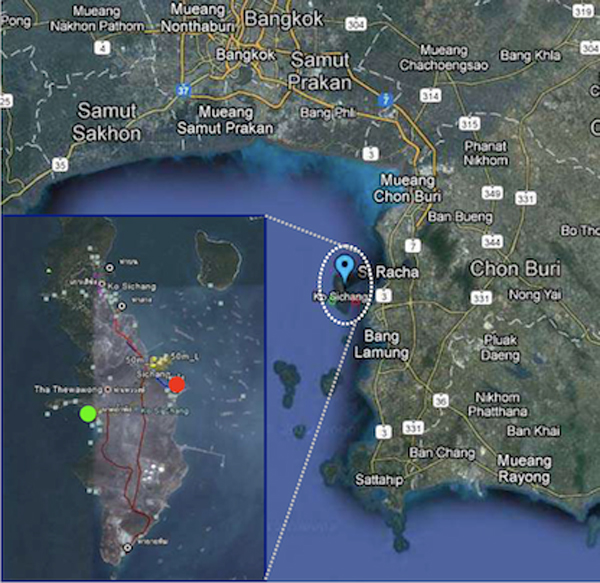
**Satellite map of Tha Wang and Tham Phang coasts, Sichang island**. Pictures were from Google Satellite Maps, retrieved on 26 April 2012, from http://www.mapandia.com/thailand/central/chon-buri/ko-si-chang/. Blue represents central of Sichang island. On the lower left corner was a zoon-in picture of Sichang island. Red and green circles represent Tha Wang and Tham Phang sampling sites.

Sichang island, or Koh Sichang, Chonburi province, Thailand, represents one potential place for massively diversified microbial biodiversity. Sichang island was originally a royal palace for King Rama IV-VI, and has been a gateway for local and international cargo transportation since 1800s. Nowadays, Sichang island serves as a historical sites for visitors, pier for merchants and related industries, and place for residents with assorted human-related activities, all of which affect water quality, aquatic species diversity and species richness in Sichang coastal water. The east and the west coasts of Sichang island pose the uniqueness in the bay geographies. Locating on the east named Tha Wang has comparatively close water circulation due to its closeness to two other islands, Khaam Yai and Prong islands, and the mainland of Chonburi province (Figure 1). Tha Wang is populated with residents, residential houses, piers, topioca starch agriculture, and shipping and fishing industries. In contrast, locating on the west named Tham Phang, also called collapsed cave beach, has more open water circulation (Figure 1). Tham Phang is minimally populated by islanders except occasional visitors, and has neither agriculture nor industry. Subsequently, more and increasing amount of wastes was reported in Tha Wang but Tham Phang beach. These included glass bottles, plastics, biodegradable garbage, metals and hazardous materials (S. Rungsupa, personal communication) [5]. More abundant and species-diverse of crabs were reported on Tha Wang (Shannon's diversity index = 0.895, Margalef's species-richness index = 4.346) than Tham Phang beaches (Shannon's diversity index = 0.141, Margalef's species-richness index = 0.991) because of the more deposition of organic matters from Tha Wang's wastes that could serve as food sources for the crabs (S. Piyatiratitivorakul and S. Rungsupa, personal communications and unpublished data).

Presently, < 1% of microbiota has been discovered, primarily owning to the limited cultivation ability and limited NCBI databases [6,7]. Culture-independent approach was first proposed by Norman R. Pace and colleagues [6]. Global ocean sampling exploration (GOS) was launched in 2003 by Craig Venter to gain understanding of prokaryotic genomes and diversity for whole marine environments, including coastal water, open ocean, seafloor and seawater at different depths, starting from Sargasso Sea to West Coasts and open oceans of the United States, Baltic, Mediterranean, and Black Seas, for examples [1,3,4,8,9]. Indeed, ocean accounts for approximately 360,000,000 m^2 ^(~71%) of the earth surface, and serves as the largest bioproductive resources. Consequently, tremendously new species of bacteria have been discovered, and much information on microbial biodiversity in marine ecosystems has been unveiled by metagenomics.

This study used metagenomics combined with 16S and 18S ribosomal DNA sequencing, and represented the first to identify the biodiversity of free-living archaea, bacteria and small eukaryotes in coastal areas of Sichang island. Each sample site comprised three independent seafloor and seawater sample collections as guided by SMaRT scientists to most represent the overall sampling collections of each coastal area; besides, these two coastal areas are not that vast. Different prokaryotic and eukaryotic species and their species distributions in Tha Wang and Tham Phang coastal water were analyzed in associated with their differential characteristics in the bay geographies and manmade activities. Our prokaryotic databases were also compared against various GOS databases to better understand the ecosystem of Sichang coasts, and obtain the comprehensive picture of the entire marine ecosystems. Hopefully, our study helps enhance knowledge on the global marine ecosystems, supporting the GOS exploration.

## Results

### Different coastal characteristics of Sichang island

Additional File 1 described general water properties of Tha Wang and Tham Phang. The water temperatures were roughly equal as the two sites are only 0.010° latitude and 0.012° longitude apart. The minor increase in water temperature of 0.7°C in Tham Phang was likely because of an atmosphere temperature that arose in the afternoon as the Tham Phang water sampling was conducted after the Tha Wang water sampling. Supportively, the monthly temperature for Tha Wang and Tham Phang coastal waters in February, 2010, as measured by Sichang Marine Science Research and Training Station (SMaRT; Chulalongkorn University, Thailand) were both 30°C. Average yearly temperature of Tha Wang and Tham Phang coasts for the year 2010 were 29.55 and 29.48°C (S. Rungsupa, personal communication and unpublished data).

However, compared with Tham Phang, Tha Wang coast had more opaque water color, less salinity and pH, and high conductivity (Additional File 1). These differences, together with the facts that Tha Wang coast possess burdens by the relatively close bay geography and the growing residents and industries, suggested the differences in the microbial biodiversity was possible.

### Metagenomic DNA isolation of Tha Wang and Tham Phang coasts

Particles and organisms of larger than ~30 micron in diameter size were prohibited by the primary filtration using 4-layer sterile cheesecloth. The 4-layer cheesecloth was measured the gap size to be 30 μm in average (unpublished data). By passing the primary-filtrated water through a 0.45-micron sterile filter paper, the microorganisms of sizes ranges 0.45 to 30 μm were collected and their total genomes were isolated. Triplicate water sampling and independently triplicate metagenomic DNA extraction experiments were performed per site; the metagenomic DNA concentrations for Tha Wang and Tham Phang were 0.50 and 0.40 ng/ml of seawater, respectively (Additional file 2).

### Prokaryotic diversity of Tha Wang and Tham Phang coasts

Libraries of Tha Wang and Tham Phang-tagged 16S rDNA sequences were successfully constructed and sequenced. Of 29,739 reads, 3,541 reads (11.91%) were initially removed from an analysis as they were < 100 bases in length, leaving 17,404 reads for Tha Wang and 8,794 reads for Tham Phang. All the reads have base length within accepted read lengths by the 454 GS/FLX sequencer, and the median read length was 404 bases. With default thresholds by VITCOMIC (E-value ≤ 1E-08, similarity ≥ 80%, alignment length ≥ 50 bp) [10], 17,381 reads for Tha Wang and 8,763 reads for Tham Phang had sequence homologies to nucleotide sequences in NCBI non-redundant database [10,11]. Additional 6 reads for Tha Wang (Proteobacteria 6 reads, Actinobacteria 2 reads, Chloroflexi 1 read) and none for Tham Phang were identified using 1E-08 < E-value ≤ 1E-02. 17 reads for Tha Wang (0.098%) and 31 reads for Tham Phang (0.353%) remained unidentified 16S rDNA reads due to non-significant E-values.

**Figure 2:**
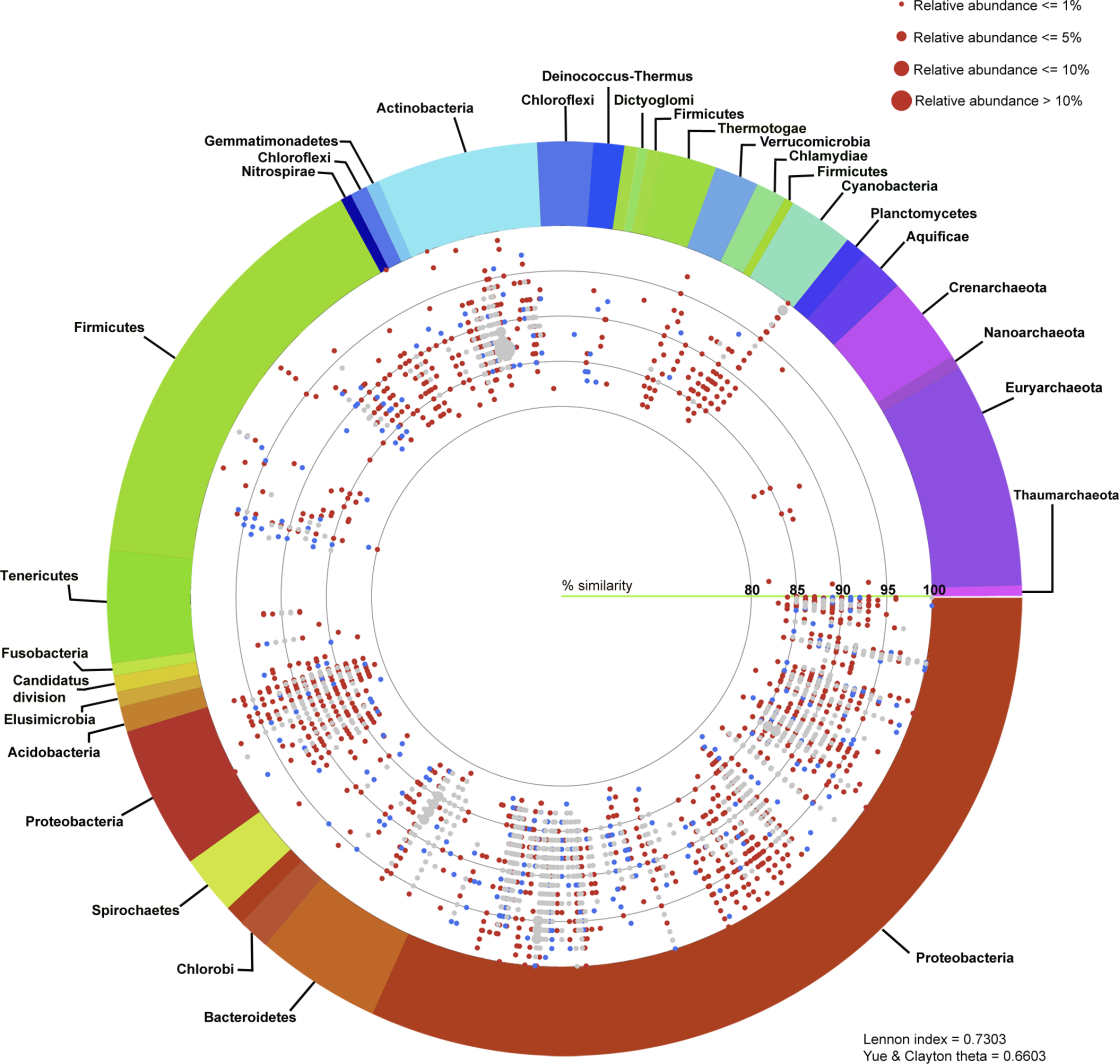
**Schematic diagram of free-living prokaryotic phyla at Tha Wang and Tham Phang coasts**. Each identified read, representing by a red (specific to Tha Wang), blue (specific to Tham Phang), or grey (both at Tha Wang and Tham Phang) dot, is annotated to species level by BLASTN with E-value ≤ 1E-08 against NCBI non-redundant databases. Pale red and blue color dot represents inclusion of significant hits with 1E-08 < E-value ≤ 1E-02. The position of each species-annotated dot on a circular diagram is arranged based on its phylogenetic distance among one another in a clockwise manner, and species belongin to the same phyla is placed the same colors. Dot size refers to a percent relative abundance as more than one read could be annotated the same species. See Methods for details.

**Figure 3:**
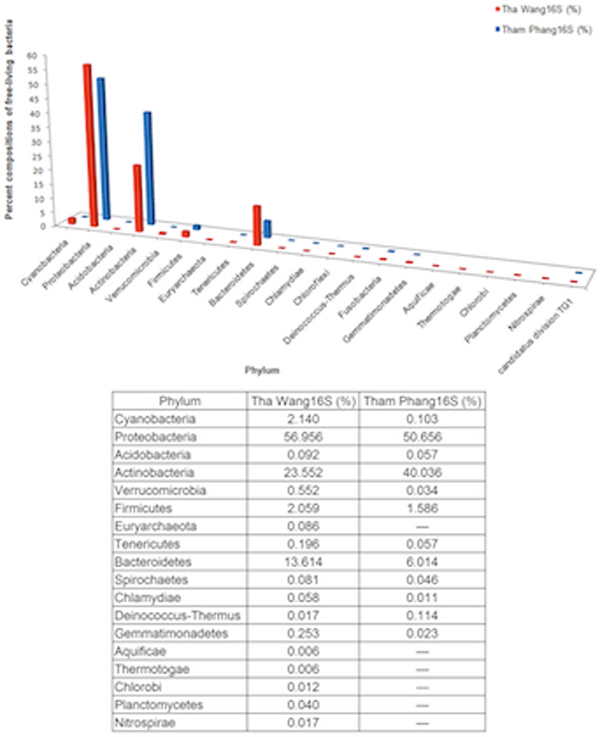
**Percent compositions of free-living prokaryotic microorganisms in Tha Wang and Tham Phang coasts**. Each identified read was classified into corresponding phylum. The proportional percentage of each phylum was calculated by dividing the number of the identified reads in a phylum with the total number of the identified reads. Tha Wang and Tham Phang were signified by red and blue barcharts, respectively.

Species diversity and relative species abundance of the identifiable reads were displayed in Additional files 3: A and B. RDP [8,12] and Greengenes [13] databases were included to generate the broader databases for species annotation, and the results were similar to Additional files 3: A and B (unpublished data). Whilst the two coasts shared many natural species and pattern of species distribution, more diversified prokaryotic species were denoted in Tha Wang (Figure 2, and Additional file 3: C). Meanwhile phylum Proteobacteria was dominant in both areas, many other phyla, including Bacteroidetes, Cyanobacteria, Firmicutes, Verrucomicrobia, Gammatimonadetes, Tenericutes, Acidobacteria, Spirochaetes and Chlamydiae were enriched in coastal water of Tha Wang than Tham Phang (Figure 3). Phyla Euryarchaeota, Planctomycetes, Nitrospirae, Chlorobi, Thermotogae and Aquificae were present only in Tha Wang coast (Figure 3). Examples of free-living prokaryotic species specific to Tha Wang coast were *Thermodesulfovibrio yellowstonii, Herpetosiphon aurantiacus, Petrotoga mobilis, Thermotoga neapolitana, Thermotoga petrophila, Halothermothrix orenii, Microcystis aeruginosa, Rhodopirellula baltica, Aquifex aeolicus, Methanopyrus kandleri, Methanococcus aeolicus, Methanoculleus marisnigri, Methanocorpusculum labreanum, Methanothermobacter thermautotrophicus *and *Aster yellows *(Additional file 3: C). For Tham Phang, species in fewer phyla were denoted (Figure 2), and phyla Actinobacteria and Deinococcus-Thermus were present at the higher proportion than Tha Wang (Figure 3). Lennon and Yue & Clayton theta similarity indices indicated 66.03-73.03% of similarity (Lennon index 0.7303, Yue & Clayton theta 0.6603) among prokaryotic communities between Tha Wang and Tham Phang coasts.

### Eukaryotic diversity of Tha Wang and Tham Phang coasts

Libraries of Tha Wang and Tham Phang-tagged 18S rDNA sequences were successfully constructed and sequenced. Of 109,024 reads, 6,698 reads (6.14%) were removed as they were < 100 bases in length, leaving 102,326 reads (Tha Wang 5,858 reads, Tham Phang 96,468 reads) for the analysis. All the reads have base length within accepted read lengths by the 454 GS/FLX sequencer, and the median read length was 465 bases. All the sequences were compared with NCBI non-redundant [11], EMBL [14,15] and SILVA [16] databases using BLASTN [10]. With default VITCOMIC parameters, 2,831 reads of Tha Wang and 95,961 reads of Tham Phang had homologous 18S rDNA sequences. Additional 12 and 15 reads for Tha Wang (Apicomplexa 1 read, Streptophyta 1 read, Dinophyta 9 reads, Porifera 1 read) and Tham Phang (Platyhelminthes 3 reads, Rotifera 1 read, Arthropoda 4 reads, Porifera 6 reads, Chlorophyta 1 read) were identified when the E-value was adjusted to 1E-08 < E-value ≤ 1E-02. The number of unidentified 18S rDNA reads, representing novel species, due to non-significant E-values by BLASTN, remained high for Tha Wang (3,015 reads, accounting for 51.468% of 5,858 reads) but Tham Phang (492 reads, 0.051% of 96,468 reads). These unidentified reads were excluded from the analysis.

**Figure 4:**
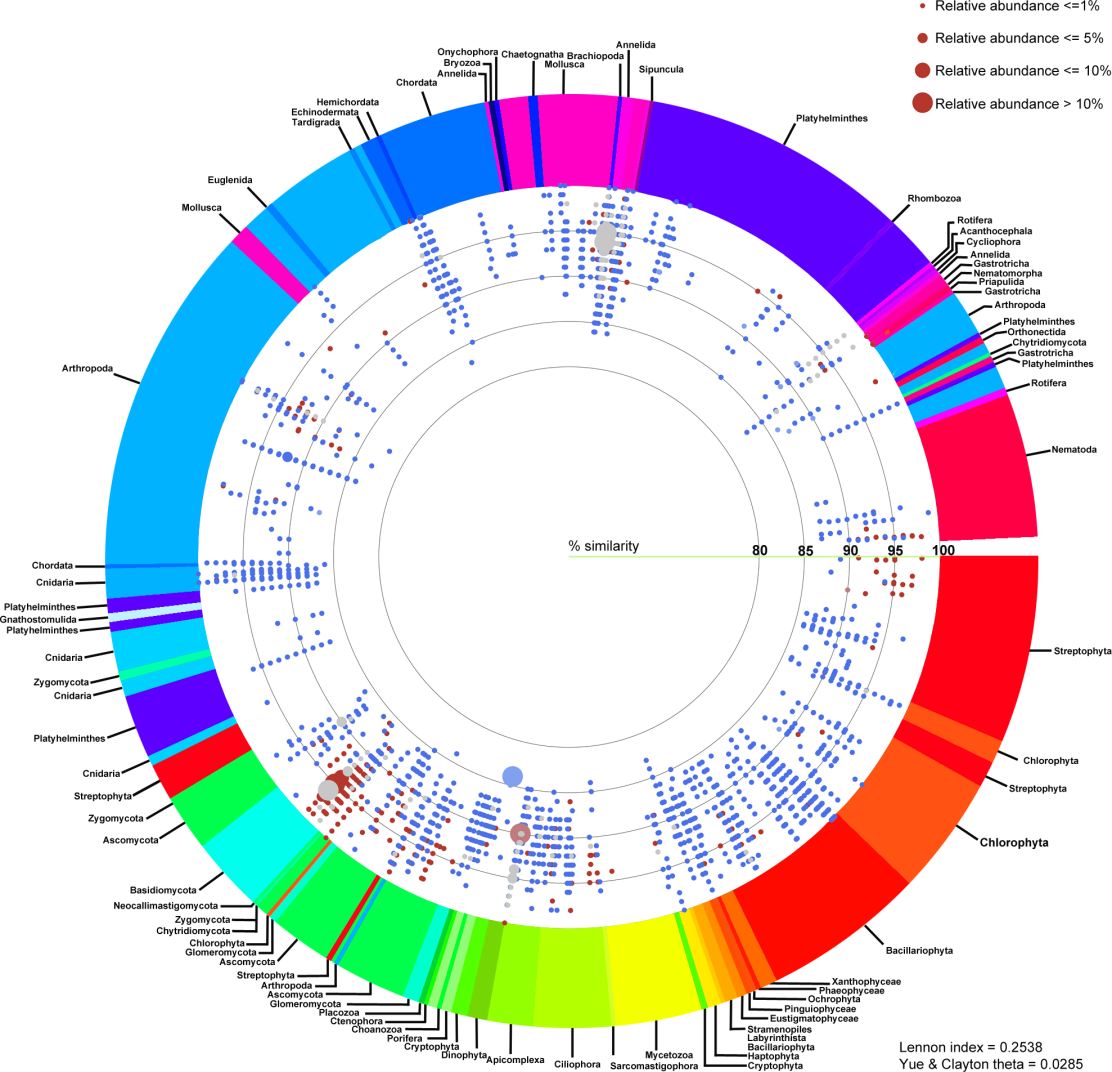
**Schematic diagram of free-living eukaryotic phyla at Tha Wang and Tham Phang coasts**. Each identified read, representing by a red (specific to Tha Wang), blue (specific to Tham Phang), or grey (both at Tha Wang and Tham Phang) dot, is annotated to species level by BLASTN with E-value ≤ 1E-08 against NCBI non-redundant, EMBL and SILVA databases. Dot color, position and size as in Figure 2. See Methods for details.

Species diversity of the identified 18S rDNA reads were displayed in Additional files 3: D and E. Unlike the prokaryotic communities, the free-living eukaryotic communities between the two coasts shared fewer similarities and more diverse species and phyla distribution pattern (Figure 4, and Additional file 3: F), highlighting by low Lennon (0.2538) and Yue & Clayton theta (0.0285) similarity indices.

**Figure 5:**
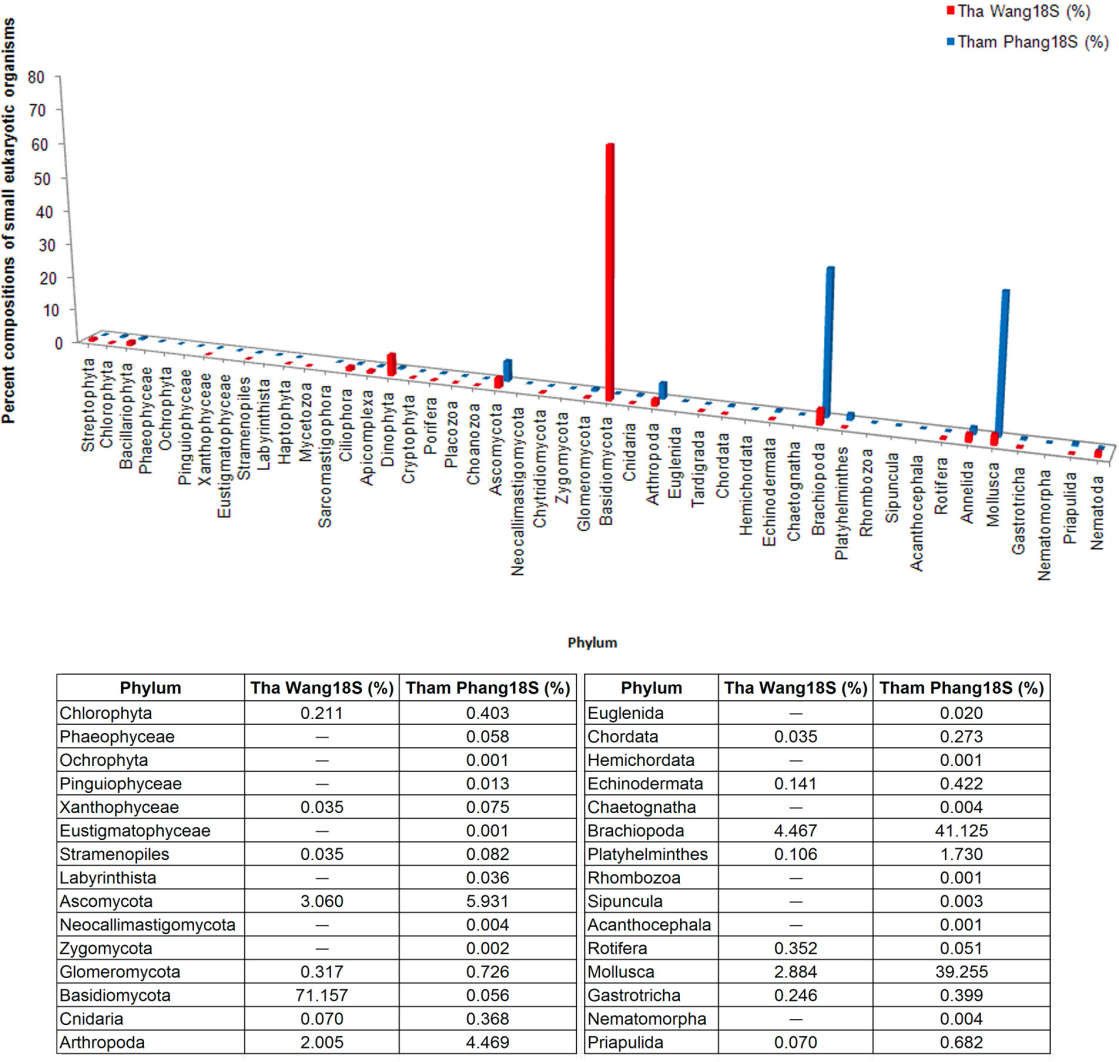
**Percent compositions of free-living eukaryotic microorganisms in Tha Wang and Tham Phang coasts**. Each identified read was classified into corresponding phylum. The proportional percentage of each phylum was calculated by dividing the number of the identified reads in a phylum with the total number of the identified reads. Tha Wang and Tham Phang were signified by red and blue barcharts, respectively. Phyla that belong to the kingdoms other than fungi are: (i) Plantae (Streptophyta, Chlorophyta, Bacillariophyta, Phaeophyceae, Ochrophyta, Pinguiophyceae, Xanthophyceae, Eustigmatophyceae, Stramenopiles, Labyrinthista, Haptophyta, Cryptophyta); (ii) Protista (Mycetozoa, Sarcomastigophora, Ciliophora, Apicomplexa, Dinophyta, Choanozoa, Euglenida); and (iii) Animalia (Porifera, Placozoa, Cnidaria, Arthropoda, Tardigrada, Chordata, Hemichordata, Echinodermata, Chaetognatha, Brachiopoda, Platyhelminthes, Rhombozoa, Sipuncula, Acanthocephala, Rotifera, Annelida, Mollusca, Gastrotricha, Nematomorpha, Priapulida, Nematoda).

While phyla Basidiomycota and Ascomycota were predominant in Tha Wang, other kingdoms of lives and species compositions were found common in Tham Phang (Figure 5). For Tha Wang, fungi were the major kingdom (74.745%), followed by kingdoms of animals (14.246%), protists (8.407%) and plants (2.603%). Tha Wang-to-Tham Phang percent composition ratios for fungi Basidiomycota, animals Tardigrada, fungi Chytridiomycota, plants Cryptophyta and protists Dinophyta were 1264.701, 33.759, 25.422, 16.879 and 13.580, respectively (Figure 5). Examples of aquatic 18S rDNA species specific for Tha Wang were *Dictyostelium deminutrivum, Penicillium oblatum, Pugettia quadridens *and *Sphyranura oligorchis *(Additional file 3: F).

In contrast, animal kingdom (91.073%), mainly phyla Brachiopoda (lamp shells) and Mollusca (snails), served the major free-living, 18S rDNA organisms detected in Tham Phang coastal water. Kingdoms of fungi (6.728%), plants (1.310%) and protists (0.890%) were present in < 10% of the identified 18S rDNA reads in Tham Phang (Figure 5). For fungi, Tham Phang coast contained proportionally more Ascomycota and Glomeromycota than Basidiomycota. Phyla belonging to other kingdoms whose percent compositions were greater than those in Tha Wang included: (i) Chlorophyta, Xanthophyceae, and Stramenopiles from the kingdom of plants; and (ii) Porifera, Cnidaria, Arthropoda, Chordata, Echinodermata, Brachiopoda, Platyhelminthes, Mollusca, Gastrotricha and Priapulida from the kingdom of animals (Figure 5). Specifically, Platyhelminthes and Mollusca held over 10% proportional abundant in Tham Phang than Tha Wang, with the Tham Phang-to-Tha Wang percent composition ratios of 16.391 and 13.610. Moreover, some phyla were only detected in Tham Phang, and they were: Phaeophyceae, Ochrophyta, Pinguiophyceae, Eustigmatophyceae, Labyrinthista, Sarcomastigophora, Neocallimastigomycota, Zygomycota, Euglenida, Hemichordata, Chaetognatha, Rhombozoa, Sipuncula, Acanthocephala and Nematomorpha (Figures 4 and 5). Examples of free-living, eukaryotic species found restricted to Tham Phang coast were *Prosopanche americana, Rafflesia keithii, Acaulospora brasiliensis, Lingula anatine, Glycymeris pedunculata, Pseudoscourfieldia marina, Hyalosira delicatula, Caloneis amphisbaena, Pinnularia acrosphaeria, Luticola goeppertiana, Leptocylindrus minimum, Chromonas cf*., *Lipomyces lipofer, Barnettozyma vustinii, Pichia salicaria, Wickernamiella domercqiae, Dipodascus magnusii, Pichia pastoris, Abies homolepis, Zschokkella hildae, Paraphanostoma crassum, Actinoposthia beklemischevi, Sphaerospora truttae, Myxobilatus gasterostei, Neoactinomyxum eiseniellae, Sterreria psammicola, Selaginopsis cornigera, Oikopleura labradoriensis, Tesserocerus dewalquei, Clavispora lusitaniae, Hyotissa numisma, Priapulus caudatus, Acantholeberis curvirostris, Eurycercus lamellatus, Ceriodaphnia megops, Malorerus randoi, Paramesopodopsis rufa, Ventsia tricarinata, Loxothylaeus texanus, Distaplia dubia, Branchiostoma floridae, Capitella sp*., *Melibe leonine, Sagitta crassa, Acanthocardia tuberculata, Ancylocoelium typicum, Scutellospora heterogama, Pteria macroptera, Peridinium balticum, Eubothrium crassum, Archotoplana holotricha, Phagocata sibirica, Paromalostomum fusculum, Chaetonotus neptuni, Bunonema franzi, Laxus cosmopolitus *and *Paracyatholaimus intermedius *(Additional file 3: F).

### Analyses of free-living prokaryotic compositions representing coastal Tha Wang and Tham Phang compared with 67 GOS profiles

Population of free-living archaea and bacteria in Tha Wang and Tham Phang coasts were compared with the 16S rDNA metagenomic profiles from 67 GOS sites. Similarity between pairs of community structures were determined by Yue & Clayton theta similarity coefficients (Thetayc) and Smith theta similarity coefficient (Thetan), using mothur [17,18]. The prokaryotic community structures between coastal Tha Wang and Tham Phang were found most closely related to each other (Thetayc 0.38756, Thetan 0.56919), and were somewhat distant from those of the GOS communities as described by the Thetayc and Thetan values that are close to 1.000 (Additional File 4). Identical community structures have zero Thetayc and Thetan value.

**Figure 6:**
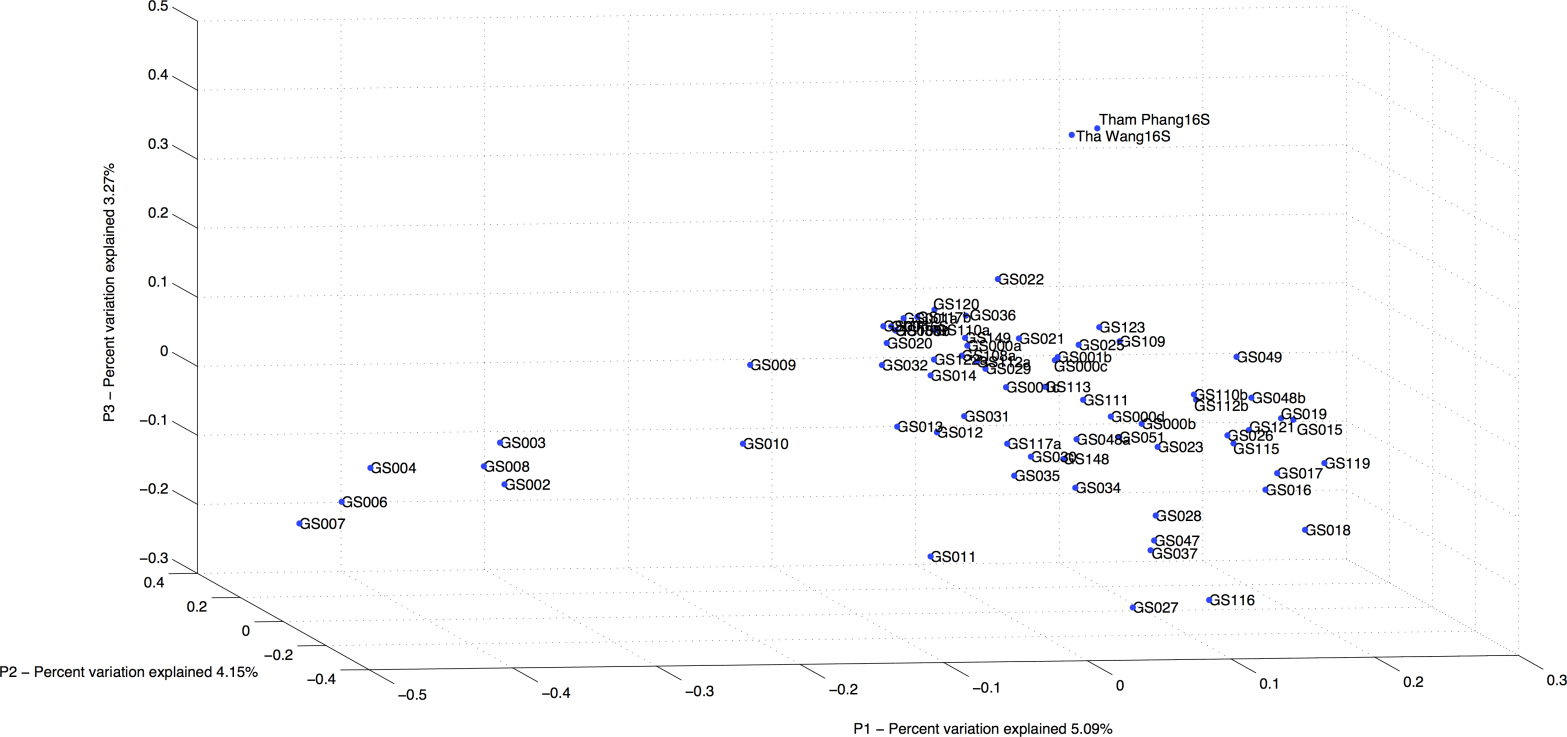
**Principle coordinate analysis comparing free-living prokaryotic compositional relatedness among Tha Wang, Tham Phang and 67 GOS sites**. Principle coordinate analysis was performed using PCoA and Thetayc in mothur [18]. The data were plotted in three dimensions.

**Figure 7:**
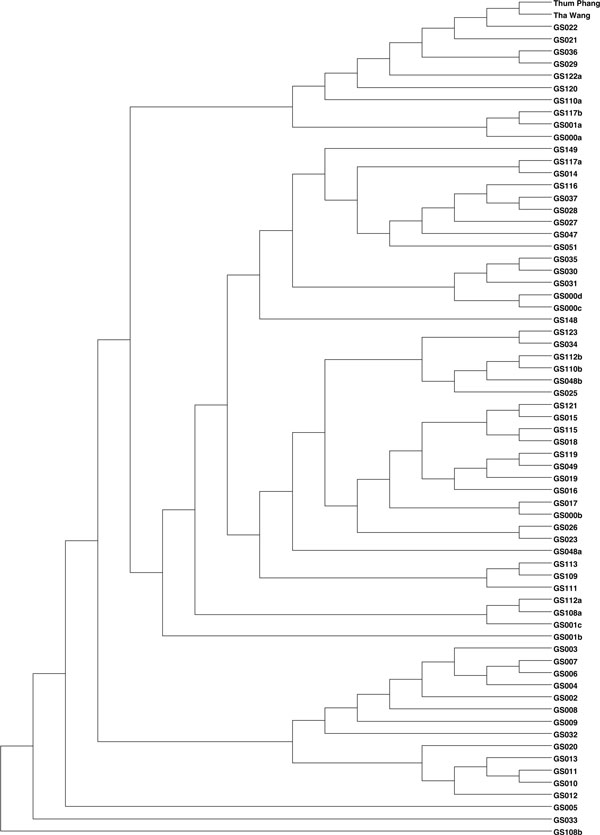
**UPGMA tree showing distances of prokaryotic communities in Tha Wang, Tham Phang and 67 GOS sites**. Distances between pairs of Tha Wang, Tham Phang and 67 GOS communities were based on the Thetayc reported in Table 2. All 16S rDNA sequences were aligned in NAST [73], following Kimura's two-parameter model [70]. The UPGMA distance tree was constructed at distance of 0.03 using mothur [18].

**Figure 8:**
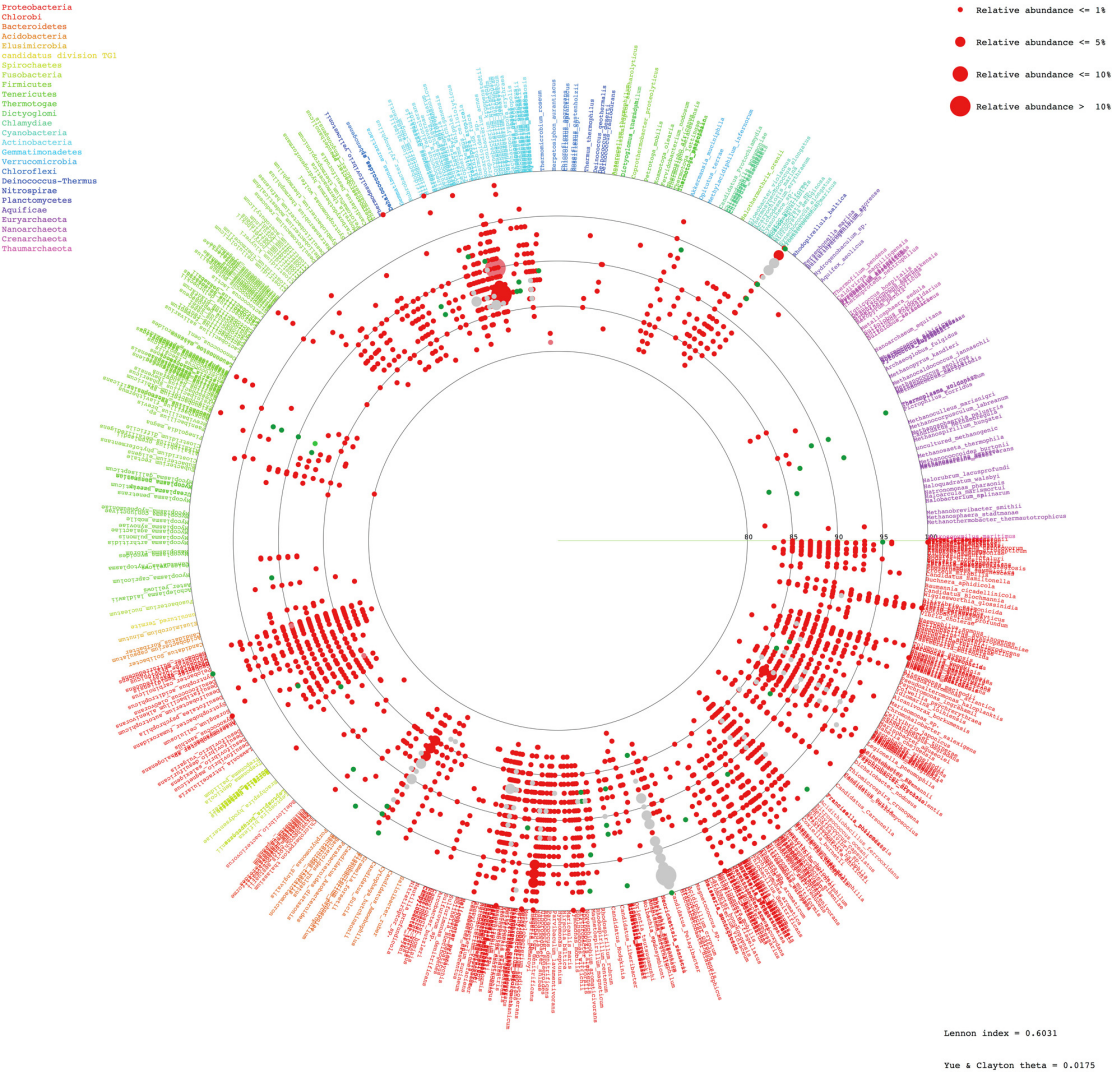
**VITCOMIC diagrams comparing prokaryotic compositions of Tha Wang against GS022**. Each identified read, representing by red (Tha Wang) or green (GS022) dot, is annotated to genus and species level with significant E-value of ≤ 1E-02 by BLASTN. Dot is placed on a circular diagram following its relative genetic distances among one another. Dot size refers to percent relative abundance as multiple reads could be annotated the same species. Grey dot represents conserved species. Different typefont color represents species belonging to different phylum. See Methods for details.

**Figure 9:**
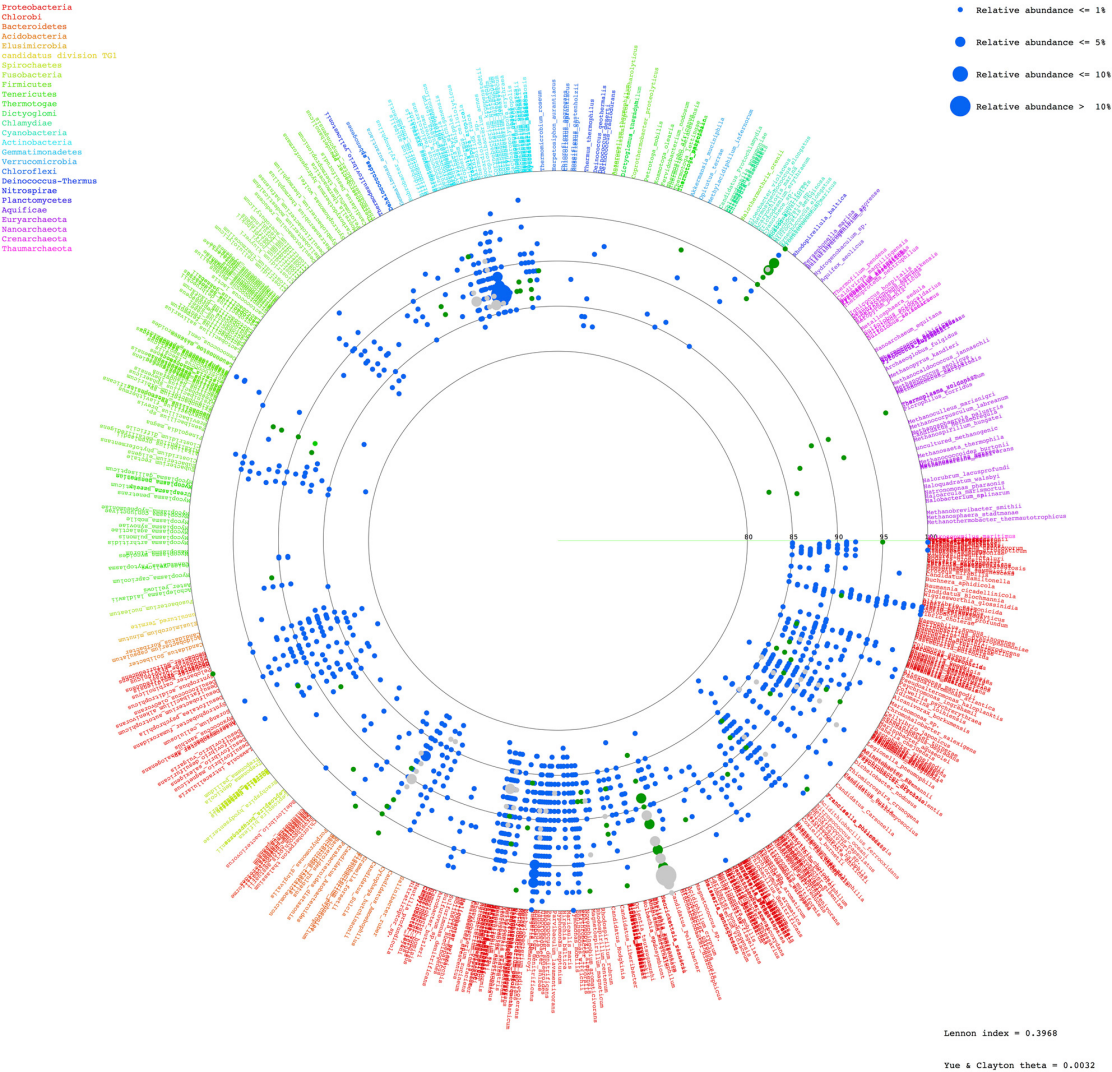
**VITCOMIC diagrams comparing prokaryotic compositions of Tham Phang against GS022**. Each identified read, representing by blue (Tham Phang) or green (GS022) dot, is annotated to genus and species level with significant E-value of ≤ 1E-02 by BLASTN. Dot position and size as in Figure 8. Grey dot represents conserved species. Different typefont color represents species belonging to different phylum. See Methods for details.

Comparing among the 67 GOS communities, the prokaryotic community structures in Tha Wang and Tham Phang coasts were most related to: GS022 (2-metre depth and 250 miles from Panama City, Panama, at latitude 6.493°N and longitude 82.904°W), GS021 (1.6-m depth from Gulf of Panama coast, Panama, at 8.129°N 79.691°W), GS028 (2-m depth from coastal Floreana, Ecuador, at 1.217°S 90.319°W), and GS015 (1.7-m depth from Off Key West coast, Florida, the United States, at 24.488°N 83.07°W) (Additional File 4). The principle coordinate analysis (PCoA) and phylogenetic tree apparently showed the prokaryotic compositions of GS022 to be most similar to those of Tha Wang and Tham Phang (Figures 6 and 7). Visualizing the prokaryotic communities among Tha Wang, Tham Phang and GS022, several conserved species as denoted by grey dots and many distance-related species were found (Figures 8 and 9). Conserved species were in phyla Proteobacteria, Actinobacteria, Bacteroidetes and Cyanobacteria (Figures 8 and 9). Comparing between Tha Wang and Tham Phang, the prokaryotic community of GS022 was closer to that in Tha Wang. The Lennon and Yue & Clayton theta similarity indices between Tha Wang and GS022 were 0.603 and 0.018, and between Tham Phang and GS022 were 0.396 and 0.003. Conserved species between GS022 and Tha Wang, and not Tham Phang were *Beutenbergia cavernae *in phylum Actinobacteria, *Halorhodospira halophila *and *Shewanella frigidimarina *in Proteobacteria, and *Prochlorococcus marinus *in Cyanobacteria (Figure 8). Conserved species between GS022 and Tha Phang, and not Tha Wang were *Rhodospirillum centenum *and *Pseudoalteromonas haloplanktis *in Proteobacteria (Figure 9). Particularly, Cyanobacteria species *Synechococcus sp*. and *Prochlorococcus marinus *in GS022 shared the great percentage of relative abundance with Tha Wang than Tham Phang. Still, some species were found restricted to GS022 and were not found in Tha Wang and Tham Phang, including *Candidatus methanoregula, Methanosaeta thermophila, Methanosarcina mazei, Methanosphaerula palustris *and *Thermoplasma volcanium *in phylum Euryarchaeota, *Geobacillus sp*. and *Lysinibacillus sphaericus *in Firmicutes, *Laribacter hongkongensis, Methylobacterium chloromethanicum *and *Rickettsia africae *in Proteobacteria, *Acholeplasma laidlawii *in Tenericutes, and *Mycobacterium avium *in Actinobacteria.

## Discussion

Tha Wang and Tham Phang coasts were hypothesized to embrace dissimilar microbial species distribution and ecosystems, given that Tha Wang represented the more inhospitable coastal niche than Tham Phang, and despite both coasts locate in similar oceanographic positions. The hypothesis was consistent with previous reports by SMaRT that found overgrowth of phytoplanktons, zooplanktons and crabs in coastal Tha Wang (S. Rungsupa, personal communication). Along with its close bay geography, the massive increase of wastes through extensive numbers of islanders and industrialization were highly responsible for the poor quality of Tha Wang bay and coast.

This study represented the first study that identified microbial biodiversity of coastal Sichang island using metagenomics, and, in consistent with the hypothesis, the study discovered different physical and chemical water properties (Additional File 1) and microbial metagenomic profiles (Figures 2 and 4) between Tha Wang and Tham Phang coasts. The clearer water, the lower conductivity and the slightly higher salinity and pH in coastal Tham Phang were appropriate for aquatic lives, and supported the diversity in marine environments and microbial communities in Tha Wang and Tham Phang coasts. The finding of the greater salinity in Tham Phang coast was coherent with the monthly salinity reports by SMaRT [19] (S. Rungsupa, personal communication). Additionally, Tham Phang coast had the greater percent dissolved oxygen and the lower contents for all kinds of wastes, including organic mass, glass bottles, plastics, metals, hazardous materials, ammonium, nitrite, and phosphate than Tha Wang coast [5] (S. Rungsupa, personal communication and unpublished data). These signified the importance for analysing the microbial diversity in Tha Wang and Tham Phang coasts, and, particularly, for investigating the effects of the relatively close bay geography and intensive manmade activities on the microbial species and species distributions in coastal Tha Wang. As Tha Wang symbolizes one central pier for Thailand's cargo route with populated residents and varied sorts of industries, yet Tham Phang remains a quiet natural beach for tourists, this further emphasized the importance of better understanding the coastal microbial ecosystems around Sichang island.

Free-living microorganisms of 0.45-30 micron in diameter size in approximate were trapped onto the filter papers, and their metagenomes were extracted. The total nucleic acid concentration of microorganisms of the two areas was averagely 0.45 ng/ml of seawater. The slightly greater metagenomic DNA concentration of free-living microorganisms in Tha Wang than Tham Phang indicated the total more amount of free-living microbiomes in Tha Wang coast (Additional file 2).

For free-living 16S rDNA species analyses, nearly 100% of the reliable read lengths (Tha Wang 99.902%, Tham Phang 99.647%) could be successfully annotated. While Proteobacteria, a major-reported bacterial phylum in global seafloor and seawater [2,4], was unsurprisingly dominated in Tha Wang and Tham Phang coasts, more diversified and abundant archaea and bacteria, including pathogenic and harsh environment-dwelling species, were revealed in Tha Wang coast (Figure 2 and Additional files 3: A-C). Many species were even found uniquely or significantly predominated in Tha Wang coast, most of which could be related to its differentially ongoing activities that polluted the marine environment and stimulated outgrowing of prokaryotic communities. For instances, Bacteroidetes are abundant bacteria in animal and human feces, and were likely carried to Tha Wang coastal water via municipal wastewater. Bacteroidetes are opportunistic pathogens to humans [20]. Cyanobacteria, also known as blue-green bacteria or algae, though are oxygen-producers and account for 20-30% of the Earth's protosynthetic productivity [21], could produce harmful cyanotoxins under constraint circumstances generally as a result of human-polluted activities [22]. *Microcystis aeruginosa *is one example. Cyanotoxins, such as neurotoxins and endotoxins, are toxic to various aquatic lives and humans consuming these contaminated water or seafood [23,24]. Firmicutes constitute a large portion of mouse and human gut microbiome, and some, such as *Clostridium *and *Bacillus*, could cause human intestinal disease [25,26]. Firmicutes could produce endospores under a hostile environment [27] like Tha Wang. Verrucomicrobia and Gammatimonadetes are new phyla that are under-represented by culture, but are believed to be common in nature, especially in soils [28]. Ternicutes refer to bacteria with no cell wall, such as *Mycoplasma *and *Ureaplasma*, that could cause human respiratory and urogenital tract diseases [27]. Acidobacter are another new phylum believed to be widespread in nature, although a few had only been isolated due to the limitation of traditional cultivation methods. The first species of Acidobacter was isolated in 1991 [29-32]. Chlamydiae are obligate intracellular bacterial pathogens that often reside asymptomatically in a variety of hosts. Chlamydiae cause severe diseases in restricted hosts, particularly humans [33]. Indeed, *Chlamydia trachomatis *is responsible for the main bacterial cause of human's preventable blindness and sexually transmitted disease worldwide [34,35]. Euryarchaeota are methane-producing archaea that survive in high-salt and high-temperature conditions.

Thus, many 16S rDNA species around Sichang island comprised archaea, which are often thermophiles, and thermophilic bacteria that could survive up to 122°C. The thermophilic bacteria were among the earliest bacteria, and include Firmicutes, Thermotogae, Aquificae, Actinobacteria, Deinococcus-Thermus and Chloroflexi [36]. Numerous archaea and thermophilic bacteria in coastal Tha Wang might be associated with tremendouse sewage drainage in Tha Wang area. Examples of species that could inhabit harsh environments and were distinctly found in Tha Wang included: sulfate-reducing *Thermodesulfovibrio yellowstonii*, originally isolated anaerobically from hot vent water in Yellowstone Lake, Wyoming, USA [37]; sulfur-reducing *Petrotoga mobilis*, obligate anaerobe that tolerates salty and high temperature and was first isolated from a North Sea oil-production well [38]; *Halothermothrix orenii*, a halophilic, thermophilic, fermentative, obligate anaerobe that could synthesize thermohalophilic enzyme and hydrogen for biotechnology [39]; *Aquifex aeolicus*, commonly found near hot springs and underwater volcanoes [40]; archaea *Methanopyrus kandleri*, originally discovered from the Gulf of California, USA, at a depth of 2000 metres [36]; and *Thermotoga neapolitana *and *Thermotoga petrophila *[39,41]. Firmicutes *Halothermothrix orenii *and all members of Thermotogae could also produce hydrogen from organic wastes, and some species such as *Thermotoga neapolitana *and *Halothermothrix orenii *could accumulate a considerate hydrogen quantity than the others [36,41]. In addition, Euryarchaeota *Methanopyrus kandleri, Methanococcus aeolicus, Methanoculleus marisnigri, Methanocorpusculum labreanum *and *Methanothermobacter thermautotrophicus *are natural carbon recycler and could produce methane from hydrogen and carbon dioxide. These archaea and bacteria are commonly detected in wastewater, sewage sludge and landfills [42,43]. Since the market demand for methane and hydrogen gases has increased yearly, using methanogenic- and hydrogenic-producing archaea and bacteria as biofuel producers represent one effective economical and environmental-sustainable venue [41].

Whereas serving as a credible resource for biofuel, Tha Wang coast also comprised many prokaryotes that could potentially cause diseases not only to humans but also to plants and other prokaryotic species in the ecosystems. For instances, *Herpetosiphon aurantiacus *in phylum Chloroflexi, which was firstly isolated from slime coat of algae from Birch Lake, Minnesota, USA [44], could secrete hydrolytic enzymes that are harmful to gram-positive and gram-negative bacteria [45]. *Aster yellows *was reported a leading cause of plant pathogen in both agricultural and nursery industries [46].

For Tham Phang, prokaryotic species advanced for biological decomposition and pharmaceutical production seemed to be more found. The relative proportion of Actinobacteria was almost 2-fold predominated in Tham Phang (41.036%) than Tha Wang (23.552%) (Figure 3). Actinobacteria are common phylum of bacteria in soil and marine environments, and have a vital role in decomposition of organic materials, such as cellulose and chitin, and of other essential nutrients [47]. Actinobacteria, such as *Streptomyces*, are also well-known for secondary metabolite producers and significant sources for pharmaceutical usage, albeit some, such as *Mycobacterium *and *Corynebacterium*, are human pathogens [48]. Interestingly, Deinococcus-Thermus, which relative proportion was also greater in Tham Phang than Tha Wang, are extremely resistant to heat, cold, anaerobic condition, and radiation materials, and they could digest nuclear and many other toxic wastes [36,49]. This phylum might further help recuperate Tham Phang coast.

The similarities and differences in 16S rDNA species diversity and species richness between Tha Wang and Tham Phang were summarized by Lennon's and Yue & Clayton theta indices of similarity (Figure 2). The lower than 74% of the similarity indices inferred a greater than 25% of difference in their prokaryotic species compositions, and supported the preliminary knowledge on Sichang geography and the results on water characteristics and 16S rDNA pyrosequencing. Moreover, comparing the 16S rDNA metagenomic profiles highlighted the greater proportions for high-temperature and energy-producing prokaryotes in Tha Wang, and for nutrient-recycling and drug-synthesizing prokaryotes in Tham Phang.

For free-living 18S rDNA species analyses, while most of the 18S rDNA reads (99.949%) could be identified with significant BLASTN E-values, approximately half of Tha Wang reads (48.532%) were identified. This suggested a large portion of distantly-related or undiscovered species in Tha Wang coast, and the research team is developing innovative bioinformatic approach to analyze these unidentified reads.

Among the identified 18S rDNA reads, Tha Wang and Tham Phang coasts shared few similarities in free-living eukaryotic compositions (Figure 4 and Additional file 3: D-F) as represented by the low Lennon and Yue & Clayton theta similarity indices. In Tha Wang, almost 75% of the identified species were fungi (Basidiomycota 71.157%, Ascomycota 3.060%, Glomeromycota 0.317%, Chytridiomycota 0.211%), and the rests comprised kingdoms of animals, protists and plants (mostly unicellular algae), in orderly (Figure 5). On the opposite, only 6.728% of the 18S rDNA reads in Tham Phang were fungi and with the different fungal compositions than those of Tha Wang. Basidiomycota and Chytridiomycota were minutely present in Tham Phang, while Ascomycota were more present. Species in animal kingdom (91.073%) were the major 18S rDNA population, and the least dominated species remained in the kingdoms of plants (1.310%) and protists (0.089%), most of which were unicellular and have chloroplasts (Figures 4 and 5). Overall, abundant population of fungi were present in Tha Wang, whereas small eukaryotes in animal kingdom were more common in Tham Phang. These differences likely reflected the differences in ecosystems between Tha Wang and Tham Phang coasts. The immense proportion of animals supported the physical and chemical characteristics of Tham Phang bay and water that was more appropriate for aquatic lives, stressing the fruitfulness of aquatic lives in Tham Phang compared with Tha Wang. Brachiopoda and Mollusca were the common species reported in Sichang island (S. Piyatiratitivorakul and S. Rungsupa, personal communications), and the frequent finding of the DNAs corresponding to these two phyla in Tham Phang was possible, as the eggs and larvae of these early developmental phase could have sizes smaller than 30 μm and thus could pass through the first filtration step. Larvae of Brachiopoda were distasteful for fish and crustaceans, and could stay in water for months; yet, they are vulnerable to pollution and were used as a measure of environmental conditions in an oil terminal in Russia and in Japan [50]. Mollusca are accounted for the largest marine phylum, and served as food and pearls for humans, so the number has been declining globally [51]. Moreover, some animals could be microscopic in sizes, such as species in Gastrotricha and Arthropoda. Meanwhile, marine fungi generally inhabited driftwood and are rare as free-living, so the high proportion of fungi in Tha Wang might be associated with the released wastes from residents and industries [52,53]. Other protists, previously considered lower fungi, were also denoted in Tha Wang, such as Mycetozoa.

The metagenomic analyses for 18S rDNA profiles in Tha Wang and Tham Phang were consistent with the analyses for the 16S rDNA profiles in that a large proportion of harsh-dwellers, including biofuel producers, were in Tha Wang, while a large proportion of decomposers and drug-producers were in Tham Phang. For examples, Ascomycota are important decomposers and medical producers [54-56], and the proportion of this phylum was found two-times in Tham Phang (Figure 5). Genera in Asocimycota are such as *Pennicilium, Tolypocladium *and *Saccharomyces*.

Moreover, the 16S rDNA metagenomic profiles inhabiting Tha Wang and Tham Phang coasts were compared to those of 67 GOS sites. High Thetayc and Thetan coefficients (Additional File 9) and no overlay of principle coordinate analysis (Figure 6) indicated unique prokaryotic ecosystems in coastal Tha Wang and Tham Phang, and emphasized the significant contribution of our 16S rDNA and 18S rDNA metagenomic profiles in fulfilling the knowledge on global marine ecosystems. The prokaryotic community structures of GS022, GS021, GS015 and GS028 were more closely to those of Tha Wang and Tham Phang coasts based on Thetayc and Thetan coefficients (Additional File 9). Principle coordinate analysis (Figure 6) and phylogenetic clustering (Figure 7) confirmed the GS022 prokaryotic community to be closest to the Tha Wang and Tham Phang prokaryotic communities (Figure 7). Some variations in phylogenetic clustering and PcoA were results of the differences in algorithms used for the PcoA and neighbour-joining tree construction. GS022 represents the prokaryotic communities inhabiting surface water in the Pacific Ocean 250 miles from Panama City, Panama, where the climate was tropical and the ecosystem was largely diverse like Thailand. Panama has tropical rain forest and temperature of 18.4-34.2°C in February (average 26.3°C). Besides, the economy of the Panama City partly depends on trading and shipping industries like Tha Wang coast, causing its marine environment to be somehow more similar to that of Tha Wang than Tham Phang (Figures 8 and 9). The Lennon and Yue & Clayton theta similarity indices between Tha Wang and GS022 were greater than those between Tham Phang and GS022. Yet, some species distinct to GS022, including human pathogens and water-quality indicators, could be associated with particular activities dominated around the Panama City. Meanwhile, uniquely found species in Tha Wang were such as *Thermodesulfovibrio yellowstonii *in phylum Nitrospirae. Although residual amount of sulphate is typical in seawater, sediment, and water rich in decaying organic material, agricultures and shipping industries could produce an excess of sulfate, leading to the presence of this microorganism in addition to other sulfate-reducing species possible. *Thermodesulfovibrio yellowstonii *could help other sulfate-reducing species in coastal Tha Wang to reduce sulfate to hydrogen sulfide and to degrade organic materials by oxidizing organic compounds or molecular hydrogen to obtain energy [36,37]. Examples of other species unique in coastal Tha Wang were *Kosmotoga olearia *in Thermotogae and *Rhodopirellula baltica *in Planctomycetes. *Kosmotoga olearia *was also present in Troll B oil platform in the North Sea, and *Rhodopirellula baltica *was also present in marine brackish Baltic sea (https://portal.camera.calit2.net/gridsphere/gridsphere). These species could also degrade complex carbohydrates in industrial wastewater, and members in Thermotogae could inhabit extreme environments, such as municipal wastewater treatment, oil production water and low-temperature bioreactors, supporting the feasibility of detecting these species in Tha Wang [36,57,58].

However, to better understand the Sichang marine ecosystems, similar analyses might be conducted at different time of the year, for example, in a different season, and at different time of the day. Once the data on 18S rDNA profiles by the GOS become available, the understanding of similarities and differences among the eukaryotic communities in coastal Tha Wang and Tham Phang against the GOS is also essential. In particular, comparative analyses of free-living fungal biodiversity help elucidate marine microbial communities, and yet many free-living fungi are obligate parasites to humans and marine organisms. Currently, we are working on identifying the biodiversity of archaea, bacteria and small eukaryotes at other significant marine and soil sites of Thailand. Such information will provide a complete database for microbiome profiles and enhance the understanding of local and global, marine and soil ecosystems.

Finally, finding of tremendously diverse species, including identified and unidentified species, showed the advantages of metagenomics in obtaining conclusive archaea, bacteria and small eukaryotic databases, helping to define the marine ecosystems. This study identified free-living prokaryotic and eukaryotic species diversity and differences in their compositions in Tha Wang and Tham Phang coasts. The study helped better understanding and better management of the marine microbial ecosystems around Sichang island, and the comparative data analyses share valuable knowledge for GOS databases. The results also suggested Tha Wang coast as potential one resource for discovery and isolation of biotechnology and industrial enzymes, and Tham Phang coast for discovery and isolation of pharmaceutical compounds. Nevertheless, all identified species in the present study merely represented the significant BLASTN hits to the identified 16S and 18S rDNA species. Hence, each annotation could mean the exact species match or the phylogenetically related species, depending on the E-values and the percent alignment coverage between the hit and the target sequences.

## Conclusions

Metagenomics allowed the culture-independent identification of 16S and 18S rDNA profiles of microorganisms residing in Tha Wang and Tham Phang coasts of Sichang island. Many similarities and differences in biodiversity and species distributions in both coasts were detected, and might be associated with the different bay geography and water conditions posed by Tha Wang and Tham Phang coasts. These metagenomic profiles helped better understanding of marine microbial ecosystems around Sichang island, and contributed supportive databases towards the world ocean metagenomic databases. Analysing the data together with the 67 GOS databases allowed the better interpretation of the global marine ecosystems.

## Methods

### Coastal water sample collection

Seafloor and seawater of Tha Wang and Tham Phang coasts (Figure 1) were collected into separated sterile glass containers on 19 February 2011, around 12:00-13:00 hrs. For each site, water color was noted, and on-site measurements for temperature, pH, salinity and conductivity were performed. The two coastal areas are not vast, and three independently replicate seafloor and seawater samples per site, as well as the positions for each water sample collection were as guided by the SMaRT scientists for the most representative sample collections of each coastal area. Note the samples belonging to the same sample site were pooled for pyrosequencing. All samples were transported on ice, stored in 4°C and were processed for the next steps within 14 days.

### Metagenomic DNA extraction and DNA quality examination

Each water sample was poured through four-layered sterile cheesecloth to remove debris and large-size organisms. Then, free-living prokaryotes and eukaryotes whose sizes were larger than 0.45 μm were collected using a sterile 0.45-micron filter (Merck Millipore, Massachusetts, USA). Total nucleic acids were isolated using Metagenomic DNA Isolation Kit for Water (Epicentre, Wisconsin, USA), following the manufacturer's instructions. The isolated metagenomic DNA should be randomly sheared and appear around 40 kb in size [59]. The extracted metagenomes were analyzed for DNA quality and concentration by agarose gel electrophoresis and A_260_/A_280 _nanodrop spectrophotometry.

### PCR generation of pyrotagged 16S and 18S rDNA sequences

For broad-range PCR amplification of prokaryotic 16S rDNAs, universal prokaryotic 338F (forward) and 803R (reverse) primers were used to amplify a 466-nucleotide sequence covering V3 and V4 regions of the 16S rRNA gene (*Escherichia coli *strain MYL-4, GenBank Accession no. HQ738475) [60-62]. The forward and reverse pyrotagged-16S rDNA primers for Tha Wang were 5'-*TCTCTGTG*ACTCCTACGGGAGGCAGCAG-3' and 5'-*TCTCTGTG*CTACCAGGGTATCTAATC-3', where italic sequences represent the tag sequences. The forward and reverse pyrotagged-16S rDNA primers for Tham Phang were 5'-*TCTACTCG*ACTCCTACGGGAGGCAGCAG-3' and 5'-*TCTACTCG*CTACCAGGGTATCTAATC-3' [63]. For broad-range PCR amplification of eukaryotic 18S rDNAs, universal eukaryotic 1A (forward) and 516R (reverse) primers were used to amplify a 560-nucleotide sequence at the 5'-end of the 18S rRNA gene (*Candida *sp. Y15 EG-2010, GenBank Accession no. HM161753) [64-66]. The forward and reverse pyrotagged-18S rDNA primers for Tha Wang were 5'-*AGAGTATG*CTGGTTGATCCTGCCAGT-3' and 5'-*AGAGTATG*ACCAGACTTGCCCTCC-3', where italic sequences represent the tag sequences. The forward and reverse pyrotagged-18S rDNA primers for Tham Phang were 5'-*ATGACTCG*CTGGTTGATCCTGCCAGT-3' and 5'-*ATGACTCG*ACCAGACTTGCCCTCC-3' [63]. For each sample, a 50-μl PCR reaction comprised 1× EmeraldAmp^® ^GT PCR Master Mix (TaKaRa, Shiga, Japan), 0.3 μM of each primer, and 100 ng of the metagenomic DNA. The PCR conditions were 95°C for 4 min, and 30-35 cycles of 94°C for 45 s, 50°C for 55 s and 72°C for 1 min 30 s, followed by 72°C for 10 min.

### Gel purification and pyrosequencing of pre-tagged 16S and 18S rDNA fragments

PCR products of about 466 (16S rDNA) and 560 (18S rDNA) nucleotides in length were excised from agarose gels, and were purified using PureLink^® ^Quick Gel Extraction Kit (Invitrogen, New York, USA). 400 ng each of pyrotagged Tha Wang and Tham Phang 16S rDNAs and 100 ng each of pyrotagged Tha Wang and Tham Phang 18S rDNAs were pooled and used for pyrosequencing on an eight-lane Picotiter plate. Pyrosequencing was performed using the 454 GS FLX system (Roche, Branford, CT).

### Sequence annotation and microbial composition analyses

Sequences were categorized based on their appended pyrotag-sequences, and sequences of less than 100 nucleotides were discarded. The sequences were annotated, visualized and computed for taxonomic compositions and evolutionary distances using VITCOMIC (Visualization tool for Taxonomic Compositions of Microbial Community) [10] with some modifications. In brief, a library for 16S rDNA species were constructed from NCBI non-redundant [11], RDP [8,12] and Greengenes [13] databases, and a library for 18S rDNA species were constructed from NCBI non-redundant [11], EMBL [14,15] and SILVA [16] databases. Each read was annotated using BLASTN [67] or RNAmmer [68] with default parameters, unless identified. Percent relative abundance for each phylum was computed from the frequency of reads in the phylum divided by the total number of identified reads. In VITCOMIC, multiple sequences were aligned following MAFFT 6.713 with default parameters [69], genetic distances were calculated following Kimura's two-parameter model [70], and a neighbor-joining tree was constructed using PHYLIP 3.69 [10,71]. Similarity indices of taxonomic compositions among different sample groups were determined based on Lennon and Yue & Clayton theta similarity indices [10,72]. Additionally, the 16S rDNA data were compared with those of the GOS (https://portal.camera.calit2.net/gridsphere/gridsphere) [1,3,8,9], using Yue & Clayton theta similarity coefficients (Thetayc) and Smith theta similarity coefficient (Thetan) in mothur [17,18]. The closer the similarity coefficient to 0.000 indicated the more similarity in community structures. PCoA was plotted in three-dimensions using mothur [18]. The Tha Wang and Tham Phang 16S rDNA data and the 67 GOS data and their Thetayc were used for an unweighted pair group method with arithmetic mean (UPGMA) clustering by mothur [18], given that their multiple sequence alignment were performed using NAST [73]. The results were manually inspected to ensure properly sequence annotation, clustering, and phylogenetic tree relationship.

## Competing interests

The authors declare that they have no competing interests.

## Authors' contributions

NS conceived of the study, collected samples, carried out molecular biology experiments, participated in and coordinated the data analysis, draft the manuscript. AA participated in study design and data analysis. AW performed data analysis. SCT carried out pyrosequencing. SDT participated in data analysis, and helped to draft the manuscript. All authors read and approved the final manuscript.

## Supplementary Material

Additional File 1 **Marine characteristics of Tha Wang coast, Tham Phang coast, and 67 GOS sites**. Geographical and chemical properties include site description, latitude and longitude, depth level, temperature, salinity in practical salinity unit (psu; formerly called parts per thousand), pH and conductivity. Information of all GOS, in numerical order based on the GOS station names, was downloaded from https://portal.camera.calit2.net/gridsphere/gridsphere. Click here for file

Additional File 2 **Agarose-electrophoretic gel showing 3 Tha Wang and 3 Tham Phang metagenomic DNAs**. Left lane is a 10 kb DNA marker where the top band is at 12 kb, and right lane is 80 ng of a 40 kb fosmid control. Click here for file

Additional File 3 **Schematic diagrams of free-living prokaryotic (A-C) and eukaryotic (D-F) species at Tha Wang and Tham Phang coasts**. For 16S rDNA reads, each identified read, representing by a red (Tha Wang) or blue (Tham Phang) dot, is annotated to genus and species level by BLASTN with E-value ≤ 1E-08 against NCBI non-redundant databases. For 18S rDNA reads, each identified read, representing by a red (Tha Wang) or blue (Tham Phang) dot, is annotated to genus and species level by BLASTN with E-value ≤ 1E-08 against NCBI non-redundant, EMBL and SILVA databases. Pale red and blue color dot represents inclusion of significant hits with 1E-08 < E-value ≤ 1E-02. In **C **and **F**, grey dot represents species found in both Tha Wang and Tham Phang. Each dot was placed on a circular diagram in respect to its species annotation. Species were arranged following their relative genetic distances among one another. Dot size refers to a percent relative abundance as more than one read could be annotated the same species. Different typefont color represents species belonging to different phylum. See Methods for details. Click here for file

Additional File 4 **Similarity coefficient values representing relatedness among pairs of prokaryotic community structures of Tha Wang, Tham Phang and 67 GOS**. Different pairs of the 16S rDNA metagenomic profiles belonging to coastal Tha Wang, coastal Tham Phang, and 67 GOS were compared via Thetayc and Thetan similarity coefficients. Thetayc and Thetan were computed using mothur with default parameters and at distance of 0.03 [18]. The results were ranged in the order starting from the most closely related to the farthest related pairs of communities. Identical community structures have Thetayc and Thetan equal to zero. Click here for file
