# Supplementary material for: Metagenomic profiles of free-living archaea, bacteria and small eukaryotes in coastal areas of Sichang island, Thailand
Source: BMC Genomics. 2012 Dec 7;13(Suppl 7):S29. doi: 10.1186/1471-2164-13-S7-S29 (PMC3521234; doi:10.1186/1471-2164-13-S7-S29)
Supplement: Additional File 1 — Marine characteristics of Tha Wang coast, Tham Phang coast, and 67 GOS sites. Geographical and chemical properties include site description, latitude and longitude, depth level, temperature, salinity in practical salinity unit (psu; formerly called parts per thousand), pH and conductivity. Information of all GOS, in numerical order based on the GOS station names, was downloaded from https://portal.camera.calit2.net/gridsphere/gridsphere. [file 1471-2164-13-S7-S29-S1.pdf]

Station name	Site description	Latitude (˚N)	Longitude (˚E)	Depth (m)	Temperature (°C)	Salinity (psu)	pH	Conductivity (mV)
Tha Wang coast	<1 metre from Tha Wang coast, Chonburi, Thailand	13.094	100.495	0	29.7	32.3	7.03	72
Tham Phang coast	<1 metre from Tham Phang coast, Chonburi, Thailand	13.084	100.483	0	30.5	32.8	7.39	66
GS000a	Sargasso Station 11, Bermuda	31.175	-64.324	5.0	20.5	36.7	N/A	N/A
GS000b	Sargasso Station 11, Bermuda	31.175	-64.324	5.0	20.5	36.7	N/A	N/A
GS000c	Sargasso Stations 3, Bermuda	32.175	-64.010	5.0	19.8	36.7	N/A	N/A
GS000d	Sargasso Station 13, Bermuda	31.175	-64.324	5.0	20.0	36.6	N/A	N/A
GS001a	Hydrostation S, Bermuda	32.167	-64.500	5.0	22.9	36.7	N/A	N/A
GS001b	Hydrostation S, Bermuda	32.167	-64.500	5.0	22.9	36.7	N/A	N/A
GS001c	Hydrostation S, Bermuda	32.167	-64.500	5.0	22.9	36.7	N/A	N/A
GS002	Gulf of Maine, Canada	42.503	-67.240	1.0	18.2	29.2	N/A	N/A
GS003	Browns Bank, Gulf of Maine, Canada	42.853	-66.217	1.0	11.7	29.9	N/A	N/A
GS004	Outside Halifax, Nova Scotia, Canada	44.137	-63.644	2.0	17.3	28.3	N/A	N/A
GS005	Bedford Basin, Nova Scotia, Canada	44.690	-63.637	1.0	15.0	30.2	N/A	N/A
GS006	Bay of Fundy, Nova Scotia, Canada	45.112	-64.947	1.0	11.2	N/A	N/A	N/A
GS007	Northern Gulf of Maine, Canada	43.632	-66.847	1.0	17.9	31.7	N/A	N/A
GS008	Newport Harbor, RI, USA	41.486	-71.351	1.0	9.4	26.5	N/A	N/A
GS009	Block Island, NY, USA	41.091	-71.602	1.0	11.0	31	N/A	N/A
GS010	Cape May, NJ, USA	38.940	-74.685	1.0	12.0	31	N/A	N/A
GS011	Delaware Bay, NJ, USA	39.418	-75.504	1.0	11.0	N/A	N/A	N/A

GS012	Chesapeake Bay, MD, USA	38.947	-76.417	13.2	1.0	3.5	N/A	N/A
GS013	Off Nags Head, NC, USA	36.004	-75.395	2.1	9.3	N/A	N/A	N/A
GS014	South of Charleston, SC, USA	32.507	-79.264	1.0	18.6	N/A	N/A	N/A
GS015	Off Key West, FL, USA	24.488	-83.070	1.7	25.0	36	N/A	N/A
GS016	Gulf of Mexico, USA	24.175	-84.344	2.0	26.4	35.8	N/A	N/A
GS017	Yucatan Channel, Mexico	20.523	-85.414	2.0	27.0	35.8	N/A	N/A
GS018	Rosario Bank, Honduras	18.037	-83.785	1.7	27.4	35.4	N/A	N/A
GS019	Northeast of Colon, Panama	10.716	-80.254	1.7	27.7	35.4	N/A	N/A
GS020	Lake Gatun, Panama	9.164	-79.836	2.0	28.6	0.1	N/A	N/A
GS021	Gulf of Panama, Panama	8.129	-79.691	1.6	27.6	30.7	N/A	N/A
GS022	250 miles from Panama City, Panama	6.493	-82.904	2.0	29.3	32.3	N/A	N/A
GS023	30 miles from Cocos Island, Costa Rica	5.640	-86.565	2.0	28.7	32.6	N/A	N/A
GS025	Dirty Rock, Cocos Island, Costa Rica	5.553	-87.088	1.1	28.3	31.4	N/A	N/A
GS026	134 miles NE of Galapagos, Ecuador	1.264	-90.295	2.0	27.8	32.6	N/A	N/A
GS027	Devil's Crown, Floreana Island, Ecuador	-1.216	-90.423	2.2	25.5	34.9	N/A	N/A
GS028	Coastal Floreana, Ecuador	-1.217	-90.320	2.0	N/A	N/A	N/A	N/A
GS029	North James Bay, Santigo Island, Ecuador	-0.200	-90.835	2.1	26.2	34.5	N/A	N/A
GS030	Warm seep, Roca Redonda, Ecuador	0.272	-91.633	19.0	26.9	N/A	N/A	N/A
GS031	Upwelling, Fernandina Island, Ecuador	-0.301	-91.652	12.0	18.6	N/A	N/A	N/A
GS032	Mangrove on Isabella Island, Ecuador	-0.594	-91.069	0.1	25.4	N/A	N/A	N/A

GS033	Punta Cormorant, Hypersaline Lagoon, Floreana Island, Ecuador	-1.228	-90.429	0.2	37.6	63.4	N/A	N/A
GS034	North Seamore Island, Ecuador	-0.383	-90.280	2.1	27.5	N/A	N/A	N/A
GS035	Wolf Island, Ecuador	1.389	-91.817	1.7	21.8	34.5	N/A	N/A
GS036	Cabo Marshall, Isabella Island, Ecuador	-0.021	-91.198	2.1	25.8	34.6	N/A	N/A
GS037	Equatorial Pacific TAO Buoy, International	-1.974	-95.015	1.8	28.0	N/A	N/A	N/A
GS047	201 miles from F. Polynesia, French Polynesia	-10.131	-135.449	30.0	28.6	37.3	N/A	N/A
GS048a	Inside Cook's Bay, Moorea, French Polynesia	-17.476	-149.812	N/A	28.9	35.1	N/A	N/A
GS049	Outside Cook's Bay, Moorea, French Polynesia	-17.453	-149.799	N/A	28.8	32.6	N/A	N/A
GS051	Rangirora Atoll, Fr. Polynesia	-15.144	-147.435	1.0	27.3	34.2	N/A	N/A
GS048b	Inside Cook's Bay, Moorea, French Polynesia	-17.476	-149.812	N/A	28.9	35.1	N/A	N/A
GS108a	Coccos Keeling, Inside Lagoon	-12.093	96.882	N/A	25.8	32.4	N/A	N/A
GS108b	Coccos Keeling, Inside Lagoon	-12.093	96.882	N/A	25.8	32.4	N/A	N/A
GS109	Indian Ocean	-10.944	92.059	N/A	27.2	32.6	N/A	N/A
GS110a	Indian Ocean	-10.446	88.303	N/A	27.0	32.7	N/A	N/A
GS110b	Indian Ocean	-10.446	88.303	N/A	27.0	32.7	N/A	N/A
GS111	Indian Ocean	-9.597	84.198	N/A	26.4	32.3	N/A	N/A
GS112a	Indian Ocean	-8.505	80.376	N/A	26.6	32.5	N/A	N/A
GS112b	Indian Ocean	-8.505	80.376	N/A	26.6	32.5	N/A	N/A
GS113	Indian Ocean	-7.008	76.331	N/A	27.5	33.3	N/A	N/A

GS115	Indian Ocean	-4.663	60.523	N/A	27.9	33.2	N/A	N/A
GS116	Outside Seychelles, Indian Ocean	-4.635	56.836	N/A	26.2	33.1	N/A	N/A
GS117a	St. Anne Island, Seychelles	-4.614	55.509	N/A	26.4	35.5	N/A	N/A
GS117b	St. Anne Island, Seychelles	-4.614	55.509	N/A	26.4	35.5	N/A	N/A
GS119	International Water Outside of Reunion Island	-23.216	52.306	N/A	23.8	35.4	N/A	N/A
GS120	Madagascar Waters	-26.035	50.123	N/A	22.5	35.6	N/A	N/A
GS121	International water between Madagascar and South Africa	-29.349	43.216	N/A	23.1	35.4	N/A	N/A
GS122a	International waters between Madagascar and South Africa	-30.898	40.420	N/A	20.2	35.8	N/A	N/A
GS123	International water between Madagascar and South Africa	-32.399	36.592	N/A	20.4	35.8	N/A	N/A
GS148	East coast Zanzibar (Tanzania), offshore Paje lagoon	-6.317	39.009	N/A	N/A	N/A	N/A	N/A
GS149	West coast Zanzibar (Tanzania), harbour region	-6.117	39.117	N/A	N/A	N/A	N/A	N/A

N/A represents data not available.
