# Supplementary figures and images for: Metagenomic profiles of free-living archaea, bacteria and small eukaryotes in coastal areas of Sichang island, Thailand
Source: BMC Genomics. 2012 Dec 7;13(Suppl 7):S29. doi: 10.1186/1471-2164-13-S7-S29 (PMC3521234; doi:10.1186/1471-2164-13-S7-S29)

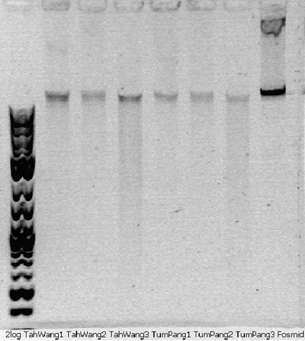

Supplement: Additional File 2 — Agarose-electrophoretic gel showing 3 Tha Wang and 3 Tham Phang metagenomic DNAs. Left lane is a 10 kb DNA marker where the top band is at 12 kb, and right lane is 80 ng of a 40 kb fosmid control. [file 1471-2164-13-S7-S29-S2.png]
