# Supplementary material for: Metagenomic profiles of free-living archaea, bacteria and small eukaryotes in coastal areas of Sichang island, Thailand
Source: BMC Genomics. 2012 Dec 7;13(Suppl 7):S29. doi: 10.1186/1471-2164-13-S7-S29 (PMC3521234; doi:10.1186/1471-2164-13-S7-S29)
Supplement: Additional File 4 — Similarity coefficient values representing relatedness among pairs of prokaryotic community structures of Tha Wang, Tham Phang and 67 GOS. Different pairs of the 16S rDNA metagenomic profiles belonging to coastal Tha Wang, coastal Tham Phang, and 67 GOS were compared via Thetayc and Thetan similarity coefficients. Thetayc and Thetan were computed using mothur with default parameters and at distance of 0.03 [18]. The results were ranged in the order starting from the most closely related to the farthest related pairs of communities. Identical community structures have Thetayc and Thetan equal to zero. [file 1471-2164-13-S7-S29-S4.pdf]

Comparing pairs		Similarity in community structure	
		Thetayc	Thetan
Tha Wang	Thum Phang	0.38756	0.56919
GS022	Thum Phang	0.85927	0.88045
GS028	Thum Phang	0.86821	0.89034
GS015	Thum Phang	0.88943	0.91162
GS021	Thum Phang	0.89441	0.95237
GS028	Tha Wang	0.89520	0.92174
GS049	Thum Phang	0.90036	0.91513
GS037	Thum Phang	0.90974	0.91049
GS029	Thum Phang	0.91182	0.91802
GS021	Tha Wang	0.91948	0.96017
GS015	Tha Wang	0.92011	0.93886
GS022	Tha Wang	0.92125	0.92591
GS049	Tha Wang	0.92878	0.93874
GS019	Thum Phang	0.93463	0.92515
GS115	Thum Phang	0.93497	0.89161
GS115	Tha Wang	0.93543	0.90944
GS051	Thum Phang	0.94341	0.91466
GS109	Thum Phang	0.94395	0.96075
GS148	Tha Wang	0.94399	0.94560
GS037	Tha Wang	0.94624	0.94317
GS036	Thum Phang	0.94742	0.94473
GS014	Thum Phang	0.94876	0.94058
GS123	Tha Wang	0.94949	0.96220
GS119	Thum Phang	0.94961	0.92744
GS123	Thum Phang	0.95101	0.95774
GS029	Tha Wang	0.95406	0.95569
GS017	Thum Phang	0.95424	0.92066
GS019	Tha Wang	0.95553	0.94931
GS014	Tha Wang	0.95746	0.95183
GS148	Thum Phang	0.95884	0.94250
GS051	Tha Wang	0.95925	0.94602
GS149	Tha Wang	0.95996	0.95134
GS000c	Thum Phang	0.96018	0.90354
GS116	Thum Phang	0.96106	0.94915
GS017	Tha Wang	0.96117	0.94573
GS109	Tha Wang	0.96199	0.97477
GS119	Tha Wang	0.96296	0.95364
GS149	Thum Phang	0.96333	0.94941

GS000c	Tha Wang	0.96416	0.93609
GS034	Tha Wang	0.96712	0.97049
GS117a	Tha Wang	0.96888	0.95176
GS018	Tha Wang	0.96896	0.96190
GS116	Tha Wang	0.97004	0.96332
GS036	Tha Wang	0.97128	0.96979
GS023	Thum Phang	0.97154	0.95920
GS001b	Tha Wang	0.97211	0.98208
GS026	Thum Phang	0.97220	0.96657
GS113	Thum Phang	0.97240	0.96087
GS018	Thum Phang	0.97383	0.95846
GS121	Thum Phang	0.97510	0.96338
GS117a	Thum Phang	0.97564	0.93564
GS000b	Tha Wang	0.97658	0.95994
GS113	Tha Wang	0.97816	0.97365
GS000b	Thum Phang	0.97922	0.94262
GS026	Tha Wang	0.97981	0.97696
GS030	Tha Wang	0.98014	0.96676
GS121	Tha Wang	0.98123	0.97484
GS023	Tha Wang	0.98132	0.97579
GS001b	Thum Phang	0.98189	0.98660
GS000d	Tha Wang	0.98198	0.96670
GS000d	Thum Phang	0.98273	0.95615
GS108a	Tha Wang	0.98394	0.98158
GS031	Tha Wang	0.98501	0.96827
GS030	Thum Phang	0.98537	0.96204
GS001c	Tha Wang	0.98546	0.98575
GS110a	Thum Phang	0.98548	0.98111
GS027	Tha Wang	0.98647	0.98229
GS025	Tha Wang	0.98949	0.99314
GS034	Thum Phang	0.99048	0.98172
GS048b	Tha Wang	0.99073	0.99029
GS111	Tha Wang	0.99082	0.98986
GS120	Thum Phang	0.99090	0.98997
GS001c	Thum Phang	0.99093	0.98728
GS027	Thum Phang	0.99131	0.98520
GS047	Tha Wang	0.99166	0.98985
GS111	Thum Phang	0.99181	0.98920
GS035	Thum Phang	0.99183	0.98587
GS016	Tha Wang	0.99185	0.98987
GS110a	Tha Wang	0.99233	0.99145

GS108a	Thum Phang	0.99251	0.98990
GS112a	Tha Wang	0.99256	0.99165
GS000a	Tha Wang	0.99264	0.97205
GS035	Tha Wang	0.99290	0.99106
GS001a	Tha Wang	0.99301	0.99162
GS000a	Thum Phang	0.99380	0.96323
GS120	Tha Wang	0.99382	0.99372
GS117b	Tha Wang	0.99382	0.99352
GS048a	Thum Phang	0.99425	0.99236
GS031	Thum Phang	0.99427	0.98260
GS016	Thum Phang	0.99448	0.99160
GS112a	Thum Phang	0.99458	0.99264
GS008	Tha Wang	0.99504	0.99183
GS047	Thum Phang	0.99510	0.99295
GS013	Tha Wang	0.99545	0.99410
GS048a	Tha Wang	0.99617	0.99528
GS008	Thum Phang	0.99663	0.99230
GS108b	Tha Wang	0.99663	0.99657
GS002	Tha Wang	0.99689	0.99668
GS006	Tha Wang	0.99736	0.99695
GS122a	Tha Wang	0.99758	0.99708
GS033	Thum Phang	0.99764	0.98851
GS032	Tha Wang	0.99787	0.99558
GS013	Thum Phang	0.99788	0.99665
GS011	Tha Wang	0.99793	0.99795
GS010	Tha Wang	0.99816	0.99771
GS033	Tha Wang	0.99823	0.99243
GS009	Thum Phang	0.99830	0.99750
GS007	Tha Wang	0.99831	0.99747
GS006	Thum Phang	0.99833	0.99818
GS003	Tha Wang	0.99833	0.99772
GS002	Thum Phang	0.99895	0.99864
GS032	Thum Phang	0.99916	0.99787
GS010	Thum Phang	0.99932	0.99898
GS009	Tha Wang	0.99938	0.99909
GS003	Thum Phang	0.99938	0.99920
GS011	Thum Phang	0.99940	0.99943
GS012	Tha Wang	0.99942	0.99931
GS122a	Thum Phang	0.99947	0.99921
GS004	Tha Wang	0.99950	0.99943
GS007	Thum Phang	0.99950	0.99966

GS004	Thum Phang	0.99959	0.99977
GS012	Thum Phang	0.99967	0.99955
GS112b	Tha Wang	0.99978	0.99983
GS005	Thum Phang	0.99980	0.99977
GS110b	Tha Wang	0.99983	0.99989
GS020	Tha Wang	0.99987	0.99960
GS005	Tha Wang	0.99989	0.99989
GS117b	Thum Phang	0.99990	0.99989
GS001a	Thum Phang	0.99992	0.99989
GS020	Thum Phang	0.99994	0.99977
GS048b	Thum Phang	1.00000	1.00000
GS108b	Thum Phang	1.00000	1.00000
GS110b	Thum Phang	1.00000	1.00000
GS112b	Thum Phang	1.00000	1.00000
GS025	Thum Phang	1.00000	1.00000
