# Supplementary material for: Metagenomic profiles of free-living archaea, bacteria and small eukaryotes in coastal areas of Sichang island, Thailand
Source: BMC Genomics. 2012 Dec 7;13(Suppl 7):S29. doi: 10.1186/1471-2164-13-S7-S29 (PMC3521234; doi:10.1186/1471-2164-13-S7-S29)
Supplement: Additional File 3 — Schematic diagrams of free-living prokaryotic (A-C) and eukaryotic (D-F) species at Tha Wang and Tham Phang coasts. For 16S rDNA reads, each identified read, representing by a red (Tha Wang) or blue (Tham Phang) dot, is annotated to genus and species level by BLASTN with E-value ≤ 1E-08 against NCBI non-redundant databases. For 18S rDNA reads, each identified read, representing by a red (Tha Wang) or blue (Tham Phang) dot, is annotated to genus and species level by BLASTN with E-value ≤ 1E-08 against NCBI non-redundant, EMBL and SILVA databases. Pale red and blue color dot represents inclusion of significant hits with 1E-08 < E-value ≤ 1E-02. In C and F, grey dot represents species found in both Tha Wang and Tham Phang. Each dot was placed on a circular diagram in respect to its species annotation. Species were arranged following their relative genetic distances among one another. Dot size refers to a percent relative abundance as more than one read could be annotated the same species. Different typefont color represents species belonging to different phylum. See Methods for details. [file 1471-2164-13-S7-S29-S3.pdf]

A.

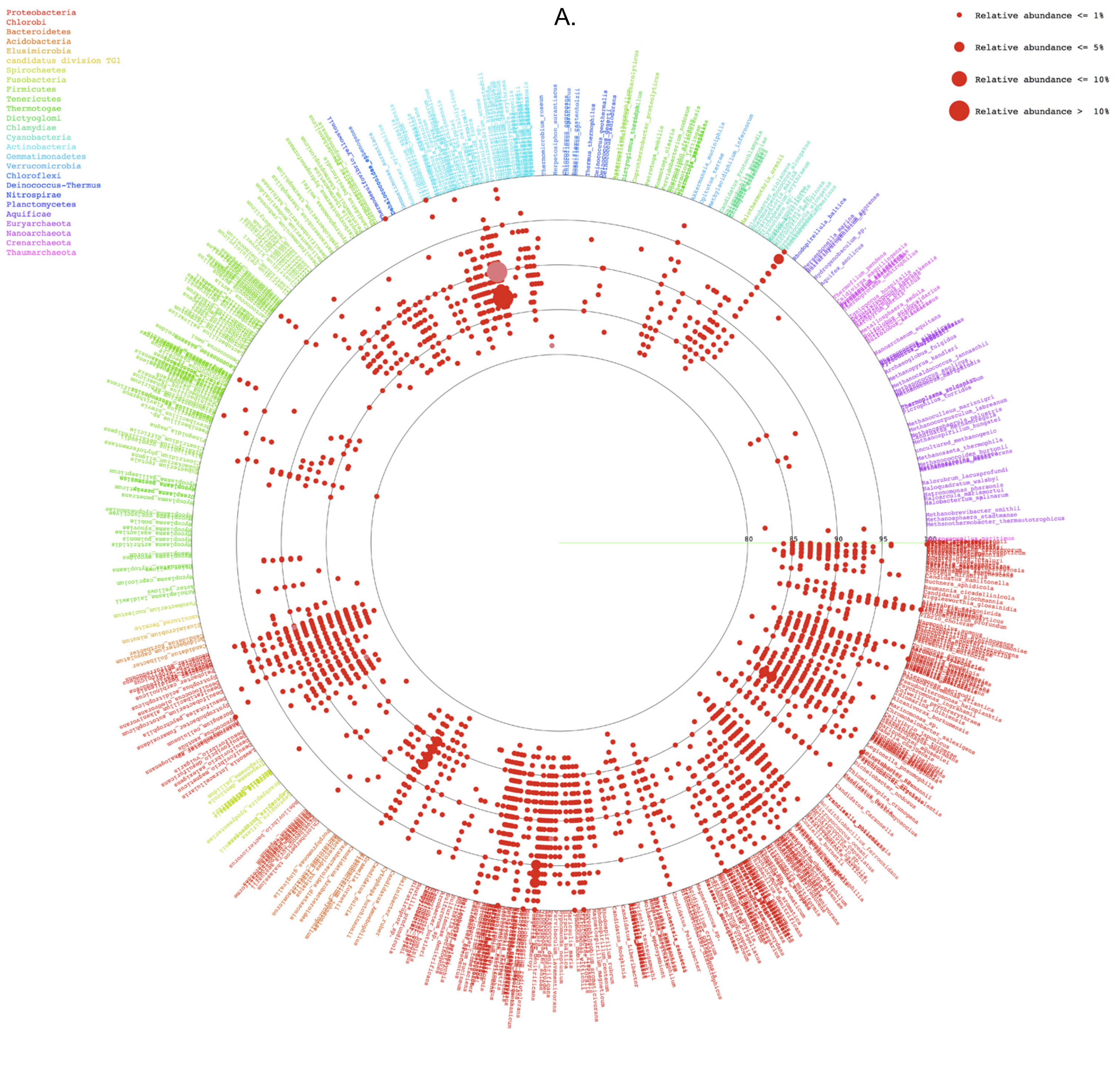

Proteobacteria
Chlorobi
Bacteroidetes
Acidobacteria
Elusimicrobia
candidatus division TG1
Spirochaetes
Fusobacteria
Firmicutes
Tenericutes
Thermotogae
Dictyoglomi
Chlamydiae
Cyanobacteria
Actinobacteria
Gemmatimonadetes
Verrucomicrobia
Chloroflexi
Deinococcus-Thermus
Nitrospirae
Planctomycetes
Aquificae
Euryarchaeota
Nanoarchaeota
Crenarchaeota
Thaumarchaeota
Relative abundance <= 1%
Relative abundance <= 5%
Relative abundance <= 10%
Relative abundance > 10%
80
85
90
95
100

B.

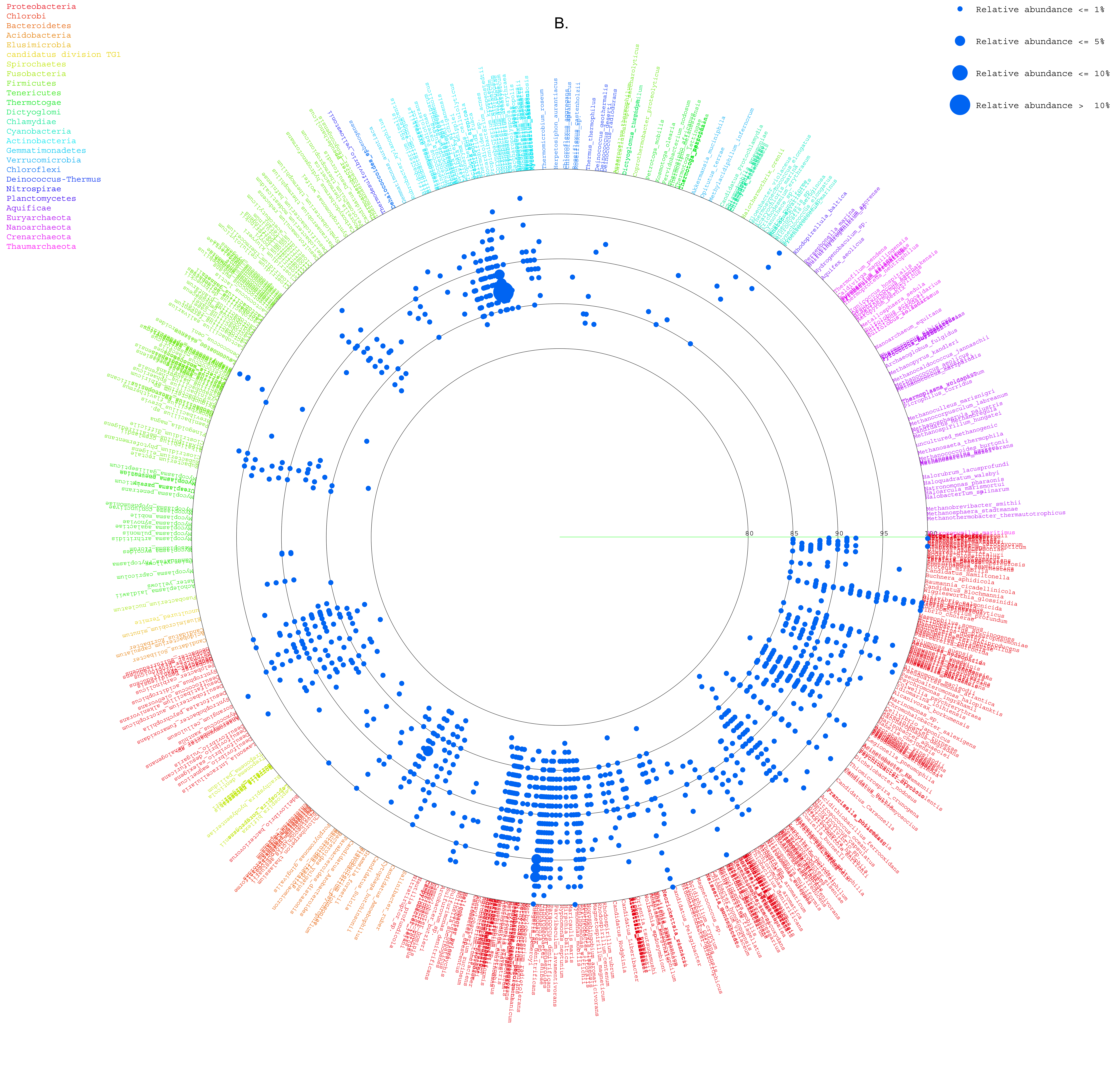
Proteobacteria
Chlorobi
Bacteroidetes
Acidobacteria
Elusimicrobia
candidatus division TG1
Spirochaetes
Fusobacteria
Firmicutes
Tenericutes
Thermotogae
Dictyoglomi
Chlamydiae
Cyanobacteria
Actinobacteria
Gemmatimonadetes
Verrucomicrobia
Chloroflexi
Deinococcus-Thermus
Nitrospirae
Planctomycetes
Aquificae
Euryarchaeota
Nanoarchaeota
Crenarchaeota
Thaumarchaeota
Relative abundance <= 1%
Relative abundance <= 5%
Relative abundance <= 10%
Relative abundance > 10%
80
85
90
95
100

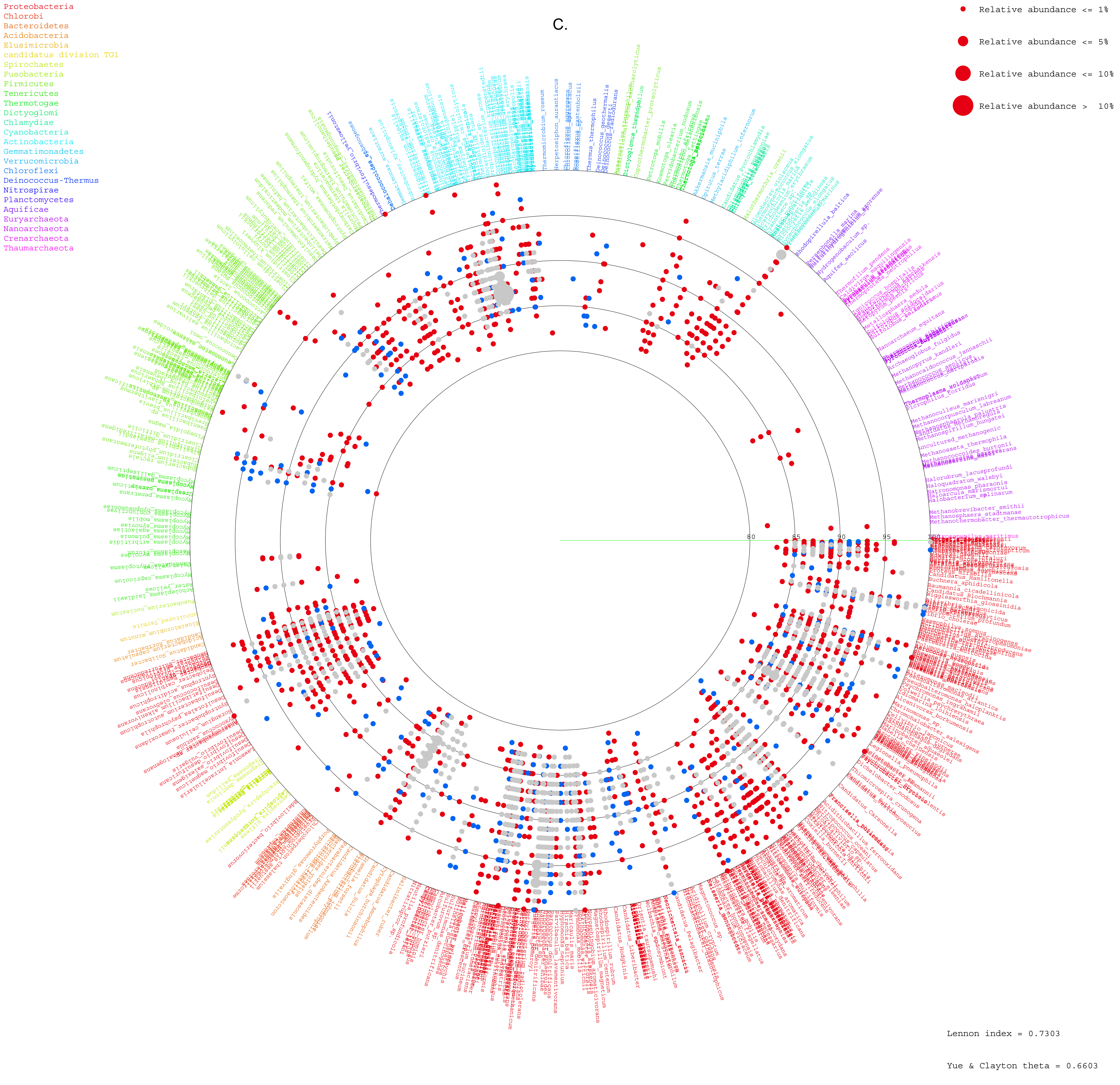

C.
Proteobacteria
Chlorobi
Bacteroidetes
Acidobacteria
Elusimicrobia
candidatus division TG1
Spirochaetes
Fusobacteria
Firmicutes
Tenericutes
Thermotogae
Dictyoglomi
Chlamydiae
Cyanobacteria
Actinobacteria
Gemmatimonadetes
Verrucomicrobia
Chloroflexi
Deinococcus-Thermus
Nitrospirae
Planctomycetes
Aquificae
Euryarchaeota
Nanoarchaeota
Crenarchaeota
Thaumarchaeota
Relative abundance <= 1%
Relative abundance <= 5%
Relative abundance <= 10%
Relative abundance > 10%
80
85
90
95
100
Lennon index = 0.7303
Yue & Clayton theta = 0.6603

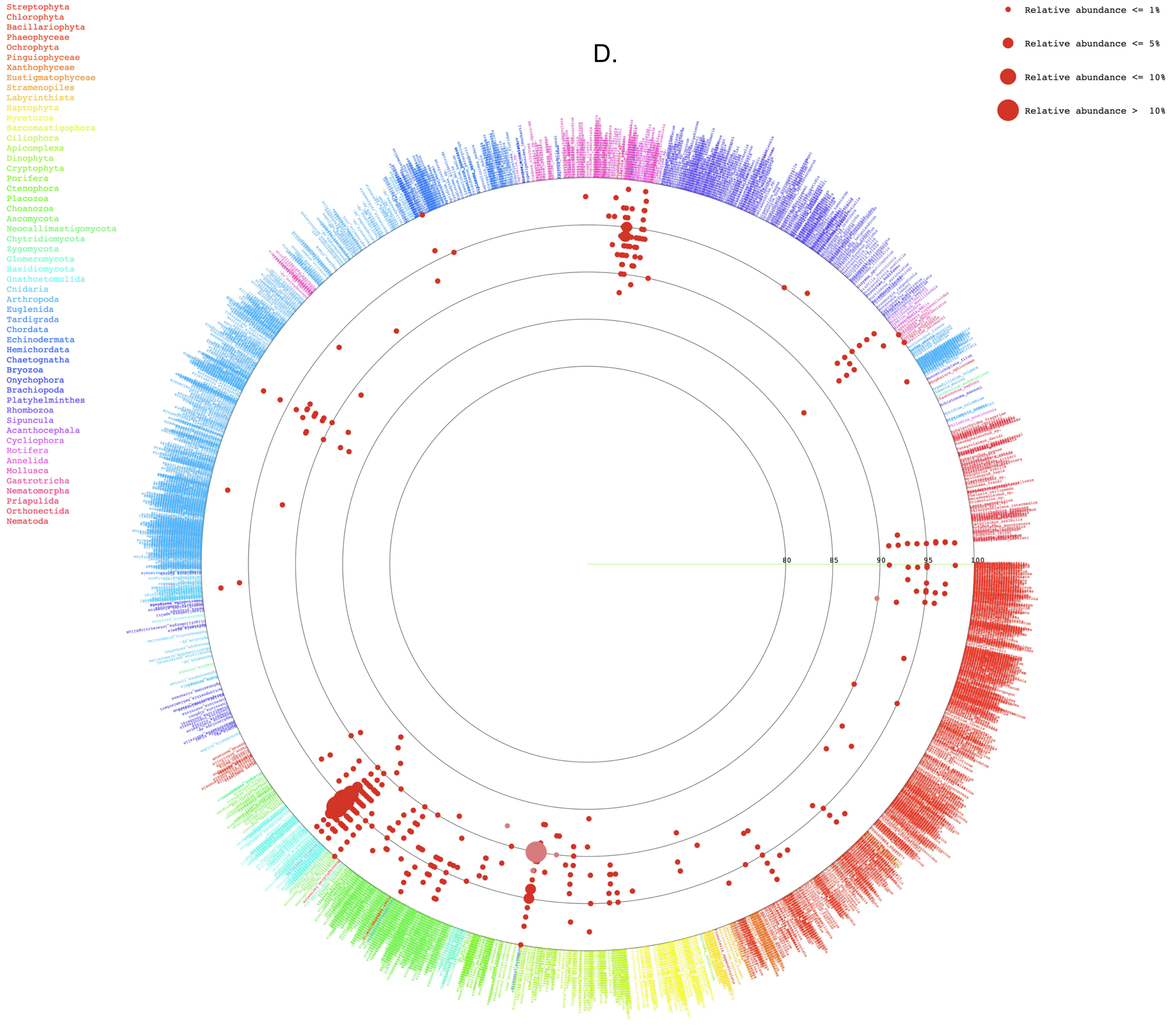

D.
Streptophyta
Chlorophyta
Bacillariophyta
Phaeophyceae
Ochrophyta
Pinguiophyceae
Xanthophyceae
Eustigmatophyceae
Stramenopiles
Labyrinthista
Haptophyta
Mycetozoa
Sarcomastigophora
Ciliophora
Apicomplexa
Dinophyta
Cryptophyta
Porifera
Ctenophora
Placozoa
Choanozoa
Ascomycota
Neocallimastigomycota
Chytridiomycota
Zygomycota
Glomeromycota
Basidiomycota
Gnathostomulida
Cnidaria
Arthropoda
Euglenida
Tardigrada
Chordata
Echinodermata
Hemichordata
Chaetognatha
Bryozoa
Onychophora
Brachiopoda
Platyhelminthes
Rhombozoa
Sipuncula
Acanthocephala
Cycliophora
Rotifera
Annelida
Mollusca
Gastrotricha
Nematomorpha
Priapulida
Orthonectida
Nematoda
Relative abundance <= 1%
Relative abundance <= 5%
Relative abundance <= 10%
Relative abundance > 10%
80
85
90
95
100

E.

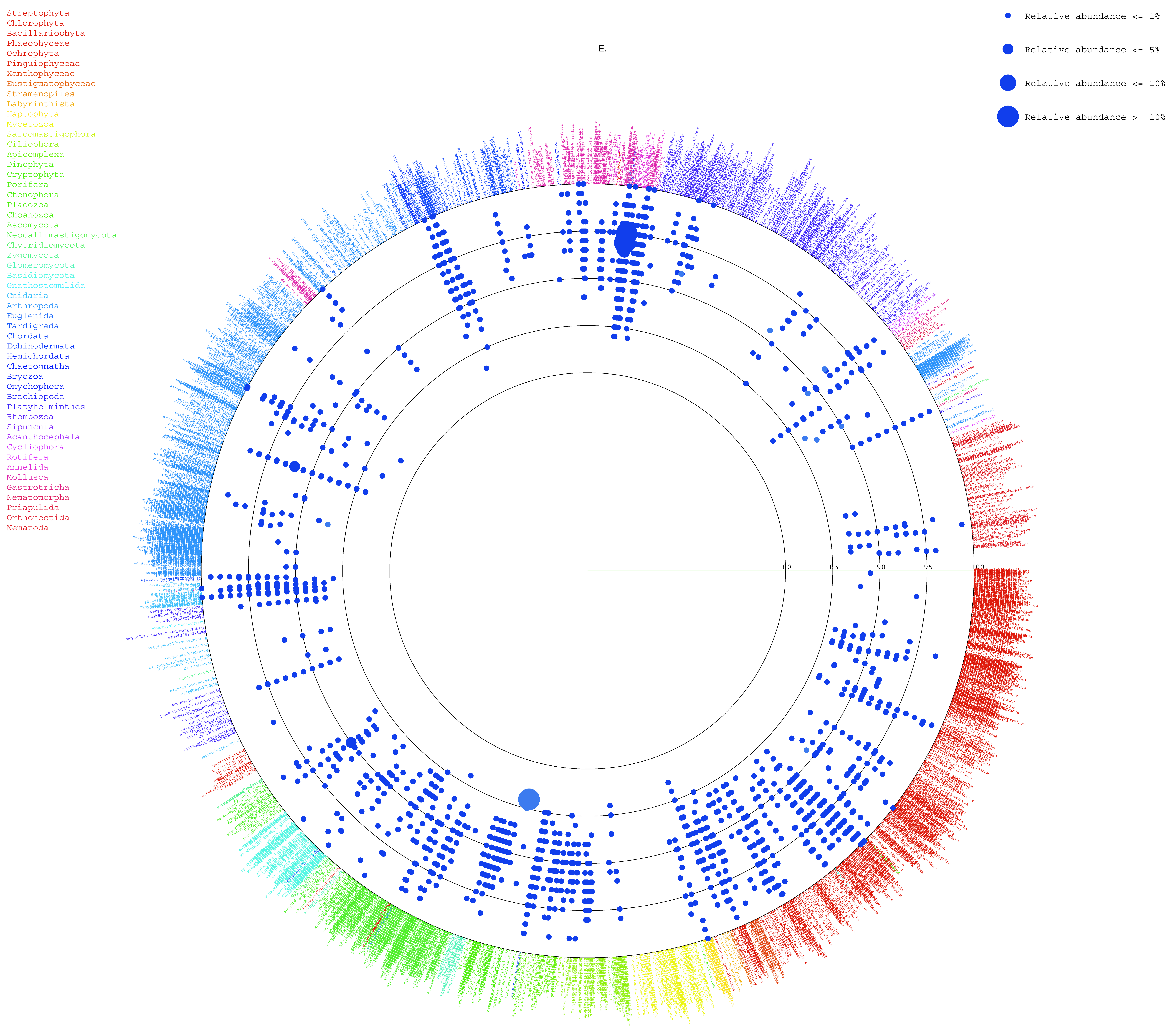
Streptophyta
Chlorophyta
Bacillariophyta
Phaeophyceae
Ochrophyta
Pinguiophyceae
Xanthophyceae
Eustigmatophyceae
Stramenopiles
Labyrinthista
Haptophyta
Mycetozoa
Sarcomastigophora
Ciliophora
Apicomplexa
Dinophyta
Cryptophyta
Porifera
Ctenophora
Placozoa
Choanozoa
Ascomycota
Neocallimastigomycota
Chytridiomycota
Zygomycota
Glomeromycota
Basidiomycota
Gnathostomulida
Cnidaria
Arthropoda
Euglenida
Tardigrada
Chordata
Echinodermata
Hemichordata
Chaetognatha
Bryozoa
Onychophora
Brachiopoda
Platyhelminthes
Rhombozoa
Sipuncula
Acanthocephala
Cycliophora
Rotifera
Annelida
Mollusca
Gastrotricha
Nematomorpha
Priapulida
Orthonectida
Nematoda
Relative abundance <= 1%
Relative abundance <= 5%
Relative abundance <= 10%
Relative abundance > 10%
80
85
90
95
100

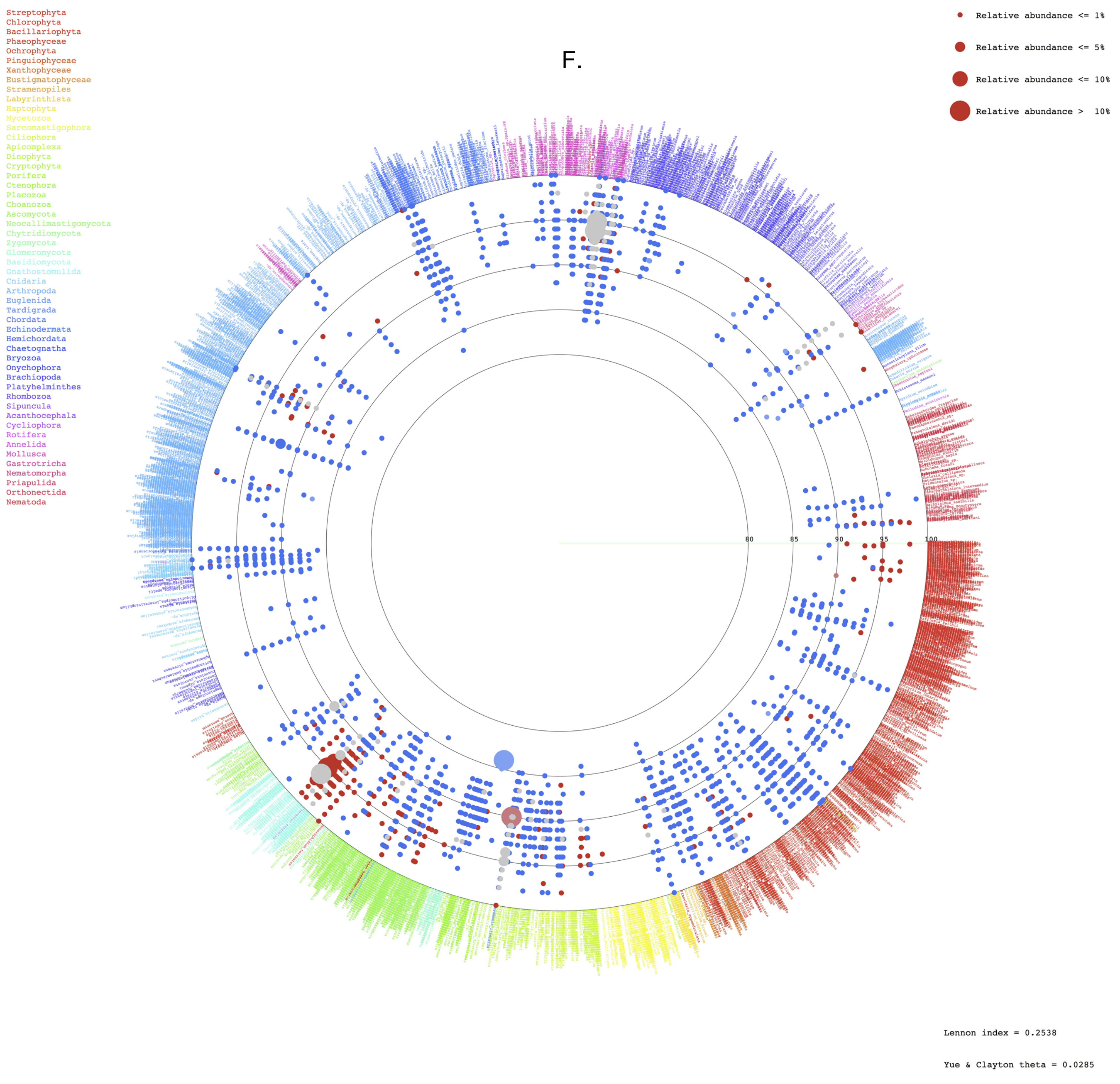
F.
Streptophyta
Chlorophyta
Bacillariophyta
Phaeophyceae
Ochrophyta
Pinguiophyceae
Xanthophyceae
Eustigmatophyceae
Stramenopiles
Labyrinthista
Haptophyta
Mycetozoa
Sarcomastigophora
Ciliophora
Apicomplexa
Dinophyta
Cryptophyta
Porifera
Ctenophora
Placozoa
Choanozoa
Ascomycota
Neocallimastigomycota
Chytridiomycota
Zygomycota
Glomeromycota
Basidiomycota
Gnathostomulida
Cnidaria
Arthropoda
Euglenida
Tardigrada
Chordata
Echinodermata
Hemichordata
Chaetognatha
Bryozoa
Onychophora
Brachiopoda
Platyhelminthes
Rhombozoa
Sipuncula
Acanthocephala
Cycliophora
Rotifera
Annelida
Mollusca
Gastrotricha
Nematomorpha
Priapulida
Orthonectida
Nematoda
Relative abundance <= 1%
Relative abundance <= 5%
Relative abundance <= 10%
Relative abundance > 10%
80
85
90
95
100
Lennon index = 0.2538
Yue & Clayton theta = 0.0285
